# Microbiome and pathobiome analyses reveal changes in community structure by foliar pathogen infection in rice

**DOI:** 10.3389/fmicb.2022.949152

**Published:** 2022-08-02

**Authors:** Khondoker M. G. Dastogeer, Michiko Yasuda, Shin Okazaki

**Affiliations:** ^1^Plant Microbiology Laboratory, Tokyo University of Agriculture and Technology, Tokyo, Japan; ^2^Department of Plant Pathology, Bangladesh Agricultural University, Mymensingh, Bangladesh

**Keywords:** amplicon sequencing, bacteria, community composition, endosphere, infection site, plant health, network analysis, source tracking

## Abstract

Increasing evidence suggests that the plant rhizosphere may recruit beneficial microbes to suppress soil-borne pathogens, but microbiome assembly due to foliar pathogen infection and ecological mechanisms that govern microbiome assembly and functions in the diseased host are not fully understood. To provide a comprehensive view of the rice-associated microbiome, we compared bacterial and fungal communities of healthy rice and those infected with *Magnaporthe oryzae*, the causal agent of blast disease. We found that the soil had a greater diversity of bacterial and fungal communities than plant endospheric communities. There was no significant dysbiosis of bacterial and fungal microbiome diversity due to disease, but it caused a substantial alteration of bacterial community structure in the root and rhizosphere compartments. The pathobiome analysis showed that the microbiome community structure of leaf and grain tissues was changed markedly at the pathogen infection site, although the alpha diversity did not change. Correspondingly, the relative abundances of some bacteria and fungi were clearly altered in symptomatic tissues. We noted an increase in *Rhizobium* bacteria and a decline of *Tylospora, Clohesyomyces*, and *Penicillium* fungi in the symptomatic leaf and grain tissues from both locations. According to the inferred microbial network, several direct interactions between *M. oryzae* and other microbes were identified. The majority of edges in the interaction network were positive in diseased samples; contrastingly, the number of edges was much lower in the healthy samples. With source tracking analysis, we observed a sharp contrast in the source of root endosphere bacteria due to *Magnaporthe* infection. Whereas the majority (71%) of healthy root bacteria could be tracked from the soil, only a very small portion (17%) could be tracked from the soil for diseased samples. These results advanced our understanding and provided potential ideas and a theoretical basis for studying pathobiome and exploiting the microbiome for sustainable agriculture.

## Introduction

The world faces formidable challenges in achieving food security for the ever-increasing population (Mc Carthy et al., 2018; Barrett, 2021). Various biotic and abiotic stressors, such as drought, salinity, disease, and pests affect crop production. In particular, plant pathogens represent an insidious threat to agriculture, and they are accounted for a loss of ~16% of global crop yield (Oerke, 2006). Numerous studies have been conducted on the intricate interplay between plant and pathogen, including pathogenicity, disease progression, plant immunity, and disease management. However, in most of these studies, the main focus of the investigation has been the binary interactions between plant and pathogen and those under various environmental conditions. The enormous complexity of interactions among plant, pathogen, and other microorganisms and their outcome under diverse conditions have also received attention in the recent years (Bulgarelli et al., 2013; Santos and Olivares, 2021; Trivedi et al., 2022).

Similar to humans and other animals, plants harbor complex microbial communities called the “plant microbiome.” There is an increasing interest in understanding the composition and function of the microbiome for harnessing their potential, such as growth promotion and disease resistance of the host, as well as in understanding the basics of host–microbial symbioses (Busby et al., 2017; Song et al., 2020; Zhang et al., 2021). Pathogenic microbes cause changes in plant phenotypes through tissue damage and induction of plant defenses, which can alter the immunity of plants to colonization by microorganisms. Therefore, factors that influence the impact of the pathogen on hosts will likely affect the colonization and growth of plant-associated microorganisms. Various host and environmental factors influence microbiome structure and diversity in the plant (Compant et al., 2019; Dastogeer et al., 2020). Host immunity level is one of the major factors shaping plant microbiome community composition (Dastogeer et al., 2020). Recent studies suggest that similar to the gut microbiome, the plant microbiome can enhance the immune functions of the plant host (Vannier et al., 2019; Ma et al., 2021; Teixeira et al., 2021). It has been shown that plants can recruit selective microorganisms from the soil by exudating compounds in the rhizosphere to positively impact plant growth and health (Reinhold-Hurek et al., 2015; Sasse et al., 2018; Liu et al., 2021).

Accumulating data implies that there remains a constant battle between the host and its microbes to maintain microbiome homeostasis in the host (Paasch and He, 2021). In the human microbiome research, the concept of “healthy microbiome” has gained massive attention, although the definition of “healthy microbiome” is still not very clear-cut (Bäckhed et al., 2012; Shanahan et al., 2021). In addition to modifying host defense, the healthy microbiome maintains ecological stability in the host and thus prevents microbial intruders, such as those protected from pathogen attack. On the other hand, the disease causes a shift in the microbiome termed, “microbiome dysbiosis,” a situation in which microbiome homeostasis is disrupted and the organism becomes more vulnerable to potentially harmful microbial invaders. The pathobiome concept arose from human studies, which suggests a shift of “one pathogen–one disease paradigm” to a set of host-associated microorganisms with reduced (or possibly reduced) health status as a result of interactions between members of that set and the host (Defazio et al., 2014; Krezalek et al., 2016). To understand the pathobiome, it is important to clarify the nature of the interactions of the associated microbes among themselves and with the host, in addition to the identity of the community (Bass et al., 2019). It was shown that perturbations in the microbial community by external factors could significantly affect the susceptibility of humans and animals to several diseases (Sekirov et al., 2010; Ferreira et al., 2011; Willing et al., 2011; Vannier et al., 2019). For example, during the progression of mastitis, dysbiosis of the milk microbiome can occur with the increase of opportunistic pathogenic bacteria and reduction of healthy commensal bacteria (Patel et al., 2017). However, it is mostly unclear whether dysbiosis is a cause or consequence of disease (Bäckhed et al., 2012). An in-depth understanding of microbiome dysbiosis may be helpful in the effort to restore a microbial community so as to abate the host damage. There is a paucity of information in the plant system regarding if and to what extent dysbiosis of the microbiome occurs due to pathogen infection in the host. In a study, Kaushal et al. (2020) showed that when *Fusarium oxysporum* infects banana plants, the fungal and bacterial communities had a shift toward a less diverse community. Humphrey and Whiteman showed that bittercress affected by a leaf-mining fly, *Scaptomyza nigrita*, had overall higher bacterial densities than undamaged leaves, mainly due to the increased abundance of typical leaf bacteria, particularly *Pseudomonas* (Humphrey and Whiteman, 2020). Increased bacterial diversity in the damaged tissue is likely caused by increased released nutrients, increased jasmonic acid, and bacterial inoculation by insect secretions (Humphrey and Whiteman, 2020; Smets and Koskella, 2020). Increased understanding of microbiome alterations under stress and functions of microorganisms in improving plant fitness and defenses highlighted the need for the investigation of their role more elaborately across various plant-pathogen-environment studies.

Rice (*Oryza sativa*) is an important crop that constitutes the staple diet of over three billion people worldwide (Skamnioti and Gurr, 2009). Rice blast disease caused by *Magnaporthe oryzae* is a global problem that can cause 10–30% reduction in rice production each year, which could feed 60 million more people (Skamnioti and Gurr, 2009; Kirtphaiboon et al., 2021). Due to a broad host range of the pathogen and the evolution of new pathotypes, blast management is a daunting task (Valent, 2021; Devanna et al., 2022). Also, environmental sustainability necessitates the innovation of natural biocontrol agents in place of chemical fungicides. Therefore, rice–*Magnaporthe* interaction pathosystem has emerged as a model system to study host–pathogen interaction. The microbiome can play a significant role in host defense and pathogen infection, and the study needs to consider pathobiome for a better understanding of the roles of disease-associated microbes. This study compared the rice microbiomes of healthy and infected rice samples gathered from the same location to gain an insight into the potential role of the pathogens in shaping the microbiome composition. We investigated the microbiome (both bacteria and fungi) structure of non-symptomatic and symptomatic rice plants naturally infected by *M. oryzae*. Symptomatic rice plants were selected based on the morphological symptoms, including typical eye-shaped brown spots in leaves and yellowish lines surrounding the spots. In addition, we demonstrated the composition and assemblage of the naturally occurring microbiome in the bulk soil, rhizosphere, root, leaf, and grain samples. We believe our study is one of the pioneer investigations to describe an inventory of bacterial and fungal communities associated with the components of non-symptomatic and symptomatic rice plants infected by *M. oryzae* in rice.

## Materials and methods

### Headings collection and processing of soil, rhizosphere, and plant samples

Rice (*Oryza sativa japonica*) samples were collected from two locations, *viz*. Fukushima and Miyagi of Japan. Samples of Miyagi (cultivar: *54-3110*) were collected from a field of Miyagi Prefectural Furukawa Agricultural Experiment Station (38.59652N, 140.91219E) on 9 September 2019, and the Fukushima samples (cultivar: *Hitomebore*) were collected from Nihonmatsu city (37.602373N, 140.586406E) grown in a farmer's field on 21 September 2019. The cultivars were susceptible to blast infection, and there were severe disease symptoms throughout the fields in both locations. In both places, samples were collected at the maturity stage of rice before the ripening phase. Rice plants were dug out with soil from around 20 cm using a shovel. We randomly chose and collected nine healthy-looking plants and nine blast-infected plants in different areas within a plot. Each plant was wrapped separately with a plastic bag and transported to the laboratory in a cooler box containing dry ice to maintain a low temperature to minimize potential microbial community disruptions. In the laboratory, the samples were further kept at 4°C until processed, and processing was completed within 48 h of collection. Sample fractionation into bulk soil, rhizosphere soil, the root, leaf, and grain compartments were performed within 48 h after sampling. At first, plants were cut above the root system. From the shoots, leaf and panicle were separated and washed with water to remove the adhering debris and named “leaf fraction” and “grain fraction,” respectively. The roots systems were shaken to collect soil manually, and the soil was labeled as “bulk soil fraction.” Large soil clods were broken with a hand-held tiller. All the debris was removed from the soil, placed in the zipper storage bag, and stored at −20°C until use. Using sterilized scissors, rice roots around 6–8 cm in length were cut. The excised roots (cut as needed to fit) were placed in a 50 ml falcon tube containing 35 ml of autoclaved phosphate buffer (6.33 g/L NaH_2_PO_4_, 8.5 g/L Na_2_HPO_4_ anhydrous, pH = 6.5, 200 μl/L silwet L-77 as surfactant). The tubes were shaken for 2 min to release the rhizosphere from the surface of the roots (rhizosphere fraction). The roots were taken out with sterilized forceps, blot dried on paper towels, and placed in a new 50 mL falcon tube (root fraction). The roots, leaves, and grains were surface sterilized by washing in 0.25% of NaOCl for 1 min, followed by 70% of EtOH for 40 s and subsequent washing in sterile water thrice. The efficacy of surface sterilization was evaluated by tissue imprint method (Greenfield et al., 2015). We used a cork borer to pierce and collect small tissues from the middle of the symptoms of leaves and named “symptomatic fraction” and from non-symptomatic tissues that did not show any apparent disease symptoms (non-symptomatic fraction). Sterilized tissues were blot dried in autoclaved paper towels, cut into small pieces, and stored at −80°C until further processed. Any plant parts or debris were removed from bulk and rhizosphere soil samples. After suspending in phosphate buffer, the soil was filtered through a sterile 100-μm-mesh cell strainer to remove any small plant parts and debris. Suspended soils were collected by centrifugation at 3000 × g for 5 min, and the pellets were stored at −20°C until DNA extraction. Rhizosphere soils and plant parts from three of the nine plants were bulked to make a biological replicate.

### DNA extraction and amplicon analysis

Total genomic DNA from bulk rhizosphere and soil was extracted using NucleoSpin^®^ Soil (Macherey–Nagel, Duren, Germany) and from the plant tissues using DNeasy^®^ Plant Mini Kit (QIAGEN, Hilden, Germany) according to respective protocols in the manuals. The DNA samples were eluted in 50 μL of nuclease-free water and used for bacterial and fungal community profiling. The quantity and quality of DNA were measured using a Nanodrop 2000, diluted to 100 ng/ml, and stored at −20°C. Bacterial 16S rRNA using 515f/806r primer pair (515f: 5′- GTGCCAGCMGCCGCGGTAA-3′; 806r: 5′-GGACTACNVGGGTW TCTAAT-3 (Caporaso et al., 2011; Apprill et al., 2015) and the fungal ITS2 using ITS1f/ITS2 primer pair (ITS1F: 5′- GTGAATCATCGAATCTTTGAA-3′; ITS2R: 5′-TCCTCCGCTTATTGATATGC-3′) (White et al., 1990; Turenne et al., 1999) were PCR-amplified. Each sample was amplified in triplicate in a 20 μl reaction volume containing 0.2 μl of Ex-Taq DNA Polymerase, 2.0 μl of 10x Ex buffer (Thermo Fisher Scientific Inc., Waltham, MA, USA), 1 μl of each primer, 13.2 μl of Milli- Q water, and 1 ng of template DNA. PCR was performed with (94°C/3 min, 94°C/45 s, 50°C/60 s, 72°C/90 s, 72°C/10 min for 35 cycles) for 16S rRNA and (94°C/3 min, 94°C/40 s, 55°C/40 s, 72°C/60 s, 72°C/7 min for 30 cycles) for ITS2. PCR quality was controlled by loading 5 μl of each reaction on a 1% agarose gel and affirming that no band was detected within the negative control. The replicated reactions were combined to make one biological replication, and the barcoded Illumina libraries were sent for paired-end Illumina MiSeq sequencing (2 × 300 bp, Bioengineering Lab. Co., Sagamihara, Japan). The obtained 16S rRNA and ITS amplicon raw reads were deposited into the Sequence Read Archive (SRA) database of the National Center for Biotechnology Information (NCBI) under the project PRJNA824966.

### Sequencing data processing and identification of amplicon sequence variants

Microbiome bioinformatics was performed with QIIME2 2020.8 (Bolyen et al., 2019). The adapter and primers were removed with Cutadapt v2.4 from the raw reads (Martin, 2011). The sequences were demultiplexed using the q2-demux plugin and followed by quality control, length trimming, denoising, chimera, and PhiX removal, and feature table construction by DADA2 with default settings except that “–ptrunc-len-f” and “–p-trunc-len-r” which were set at 250 and 200, respectively for 16S data and at 160 and 200 for ITS data, respectively (*via* q2-dada2) (Callahan et al., 2016). The resulting amplicon sequence variants (ASVs) were aligned with mafft (Katoh et al., 2002) and phylogenetic trees were constructed using fasttree2 (Price et al., 2010). Taxonomy was assigned to ASVs using the QIIME feature-classifier classify-skarn (Bokulich et al., 2018) with the pre-trained naïve Bayes SILVA classifier v132 trimmed to the V4 region of the 16S rDNA gene (Quast et al., 2012) for bacteria and pretrained UNITE ver8 99% database (UNITE Community, 2019), trained on the full reference sequences without any extraction for fungi. Non-bacterial and fungal reads were removed from the obtained ASV table. We normalized the library using scaling with ranked subsampling using the “SRS”-function in the “SRS” with “qiime srs SRS” (Beule and Karlovsky, 2020). The alpha diversity were evaluated with the chao1 estimator (richness) and the Shannon index and Faith PD in qiime2 and Kruskal–Wallis tests were used to compare the diversity. Box plots to display the alpha diversity indices were created using ggplot2 (Wickham et al., 2016) installed in R Core Team (2020). The Bray–Curtis dissimilarity matrix was calculated to assess differences in composition of bacterial and fungal communities and used for the principal coordinate analysis (PCoA) ordination plot in PAST4.04 (Hammer et al., 2001). We compared differences in community composition and structure among sampling sites (α- diversity) using the analysis of similarity (ANOSIM) (Clarke, 1993) and permutational multivariate analysis of variance (PERMANOVA) (Anderson, 2014). Least discriminant analysis (LDA) and random forest were performed using microbiomeanalyst (Chong et al., 2020).

### Functional prediction, source tracking, and network analyses of fungal and bacterial community

The marker genes in the samples (16S rRNA and ITS) were inferred for the prediction of functional profiles by Phylogenetic Investigation of Communities by Reconstruction of Unobserved States (PICRUSt2) algorithm software (Douglas et al., 2020). MetaCyc, the comprehensive reference gene function, was mapped to analyze gene functions for bacterial and fungal ASVs (Krieger et al., 2004). Pathways inferred with high-level functions were mapped against the MetaCyc database (Caspi et al., 2020). To predict the ecological function of fungi, we used the FUNGuild database (Nguyen et al., 2016), whereas, for bacteria, we used the FAPROTX (Louca et al., 2016). To estimate the association between bacterial and fungal communities in soil, the rhizosphere, roots, leaves, and grains tissues in healthy and diseased plants, we used SourceTracker2 in python (version 3.7.0) (Knights et al., 2011). We analyzed SourceTracker2 default parameters, and one by one, each environmental sample type was designated as a sink with all other environmental sample types as sources. For example, to investigate source-associated ASVs on leaves of healthy plants, root, rhizosphere soil, and bulk soil of healthy samples were specified as sources, and leaf was designated as the sink. Kruskal–Wallis tests were used to compare the mean percentages assigned to different sources. The percentage value was derived from the statistical average of the results of SourceTracker. We constructed networks of microbiome communities in healthy and infected samples using Pearson correlation with a significance of *p* < 0.05 and correlation coefficient R > 0.60. For this, we combined the ASV table of both bacterial and fungal communities. To simplify the network, we extracted only the ASVs that showed a significant correlation with *M. oryzae* and created a subnetwork of interactions between the pathogen and other microbes. Although different DNA extraction kits used for plant and soil samples might cause some variation, we considered the impact to be insignificant for our analysis.

## Results

### Soil microbial communities are more diverse and distinct from endospheric communities

**Figure 1:**
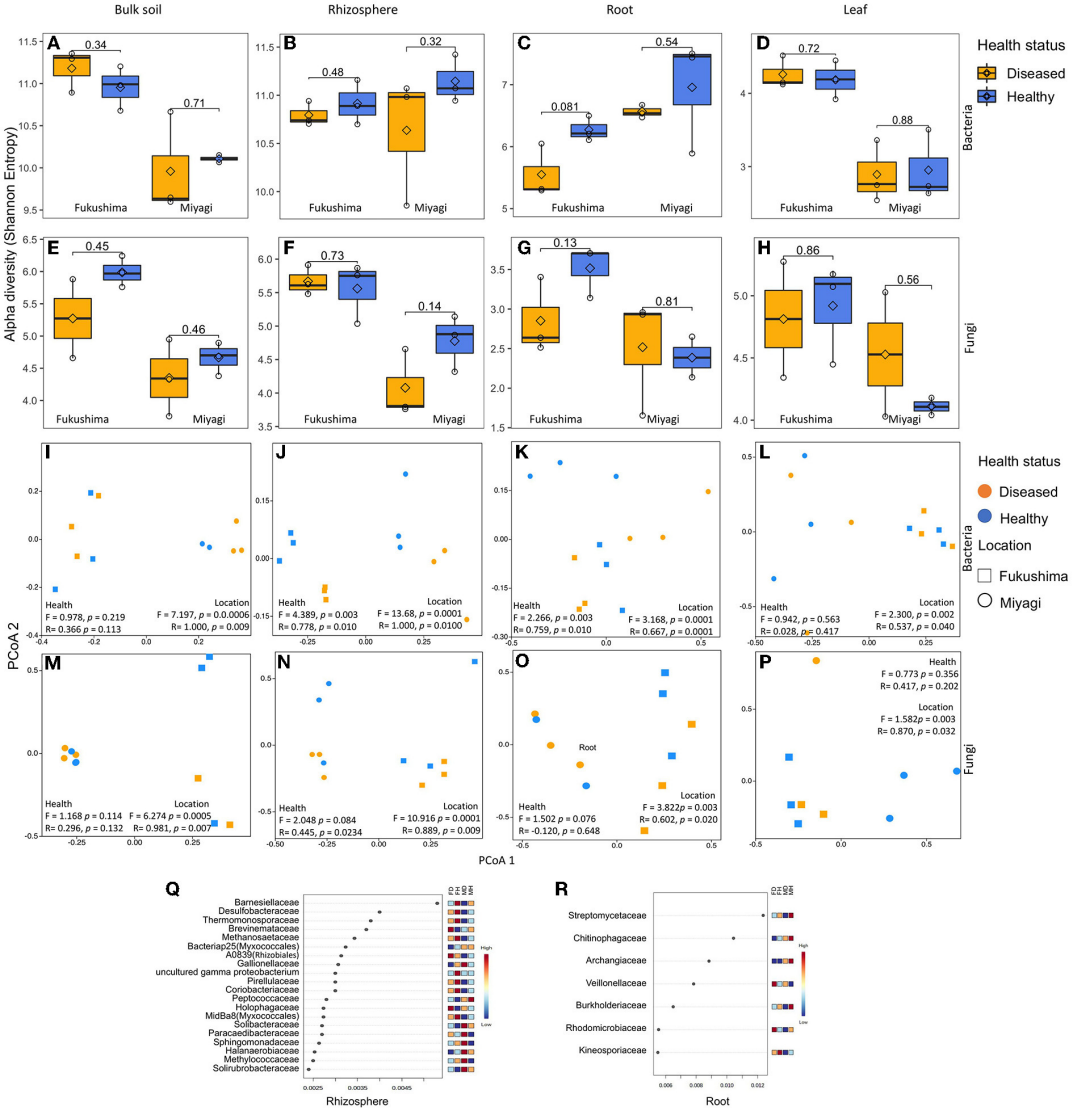
Bacterial and fungal microbiome community of rice as influenced by plant health status and location: Alpha diversity (Shannon Entropy) of bacterial **(A–D)** and fungal **(E–H)** community in bulk soil **(A,E)**, rhizosphere soil **(B,F)**, root endosphere **(C,G)**, and leaf endosphere **(D,H)**. Principal co-ordinate (PCoA), analysis of similarity (ANOSIM), and permutational multivariate analysis of variance (PERMANOVA)-based analyses of bacterial **(I–L)** and fungal **(M–P)** microbiome of healthy and diseased (non-symptomatic) samples of rice collected from two locations in Japan. Bray-Curtis coefficient of community similarity index was computed for comparing the bacterial community similarity among groups. The ANOSIM statistic *R* values (up to 1) and PERMANOVA *pseudo-F* indicate similarity/dissimilarity between groups. Ordination plots show the influence of plant health status, and location on microbial community assembly of bulk soil **(I,M)**, the rhizosphere soil **(J,N)**, root endosphere **(K,O)**, and the leaf endosphere **(L,P)**. Differential abundance of bacterial microbiome community of rhizosphere soil **(Q)** and root endosphere **(R)** rice as obtained by random forest analysis. Random forest analysis results of top 20 bacterial family with the highest discriminatory power between diseased and healthy samples of rhizosphere community are shown. Red fields show a high abundance and blue fields a low abundance of the particular bacterial family. FD, Fukushima diseased; FH, Fukushima healthy; MD, Miyagi diseased; MH, Miyagi healthy.

We analyzed and compared the alpha and beta diversity of bacterial and fungal microbiome of different compartments (i.e., soil, the rhizosphere, root, and leaf) of healthy rice plants from both locations. As expected, the rhizosphere and bulk soil microbiome were more diverse, followed by the root and leaf endophytic communities in both locations (Figures 1A–H; Supplementary Figures 1A,B). Similar patterns were observed for other diversity indices, such as Chao1 and Faith's phylogenetic diversity (PD) (Supplementary Table 1). Samples from two different locations, i.e., Fukushima and Miyagi, showed variation in the diversity of bulk soil and leaf infosphere communities (Supplementary Tables 1, 2). The PCoA revealed distinct bacterial and fungal assemblages among different compartments in the healthy samples. It was noted that bulk soil and rhizosphere microbial communities overlapped but were separated from endosphere (the root and leaf) communities which were significantly different from each other (Supplementary Figures 1C,G; (Supplementary Table 3). We checked which bacterial groups differed among compartments using LDA. With few others, Delta and Gamma-proteobacteria, Bacteroidia, Verrucomicrobiae, Acidobacteria, and Clostridia were higher in both soil and rhizosphere samples but very low or absent in plant microbiomes (root and leaf). Oxyphotobacteria is the dominant bacteria in the leaf and root microbiome, which were less abundant in the belowground microbiome (Supplementary Figure 1D). We also checked which fungal groups were different among compartments by the illustration of taxa bar plots. Eurotiomycetes, Dothideomycetes, and Agaricomycetes were more abundant in the aboveground compartment (root and leaf), whereas Sordariomycetes and unknown fungal taxa were more in bulk soil and rhizosphere communities (Supplementary Figure 1H).

### Disease-caused alteration of bacterial but not fungal community compositions in the rhizosphere and root samples

Since microbiome community composition and diversity were different in different compartments, we compared the bacterial and fungal microbiome of healthy and diseased samples separately from soil, the rhizosphere, root, and leaf. No significant difference was observed for the alpha diversity of bacteria (Shannon, Faith'sPD, and Chao1) between healthy and diseased samples regardless of the microbiome compartments (Figures 1A–D; Supplementary Table 1). PCoA, PERMANOVA, and ANOSIM analyses revealed that the rhizosphere and root compartment had distinctly different bacterial compositions in the diseased plants compared to the healthy samples (Figures 1J,K). The bacterial communities of bulk soil and leaf endophyte did not differ due to disease status. The bacterial community of the two locations was distinctly different regardless of the plant compartment. Several bacterial families were more abundant in the rhizosphere of healthy as compared to the diseased samples, such as *Barnesiellaceae, Desulfobacteraceae, Thermomonosporaceae, Methanosaetaceae, Pirellulaceae*, and *Coriobacteriaceae* in both locations. In contrast, bacteria of the families *Brevinemataceae, Holophagaceae, Paracaedibacteraceae*, and *Solirubrobacteraceae* were highly abundant in the rhizosphere communities of diseased samples (Figure 1Q). In the root communities, members of *Streptomycetaceae, Chitinophagaceae, Burkholderiaceae*, and *Kineosporiaceae* were present abundantly in the healthy samples, and *Veillonellaceae* were abundant in the diseased samples (Figure 1R). At the genus level, *Turneriella, Pelolinea, Sulfuricurvum, Desulfocapsa, Cuspidothrix*, and *Arenimonas* were more abundant in the healthy rhizosphere, and *Desulfovirga, Roseiarcus*, and *Nakamurella* in diseased samples (Supplementary Figure 2). In roots, *Streptomyces, Methylosinus, Cephaloticoccus, Burkholderia, Paraburkholderia, Caulobacter, Acinetobacter, Geothrix, Ruminiclostridium, Hydrogenophaga*, and *Pleomorphomonas* were more abundant in healthy plants in both locations (Supplementary Figure 2).

In the case of the fungal microbiome, no significant differences were observed for alpha diversity between healthy and diseased samples regardless of the microbiome compartments (Figures 1E–H; Supplementary Table 2). Fukushima samples had a higher diversity of fungal community compared to Miyagi samples which were very prominent in soil communities. However, the richness index (Chao1) did not exhibit any variation between locations except for bulk soil (Supplementary Table 2). From PCoA, we did not observe any influence of blast disease infection on the structure of the fungal community; there were locational variations in the soil as well as endospheric communities (Figures 1M–P; Supplementary Table 2).

### Pathobiome analysis of symptomatic and non-symptomatic tissue of diseased plants

**Figure 2:**
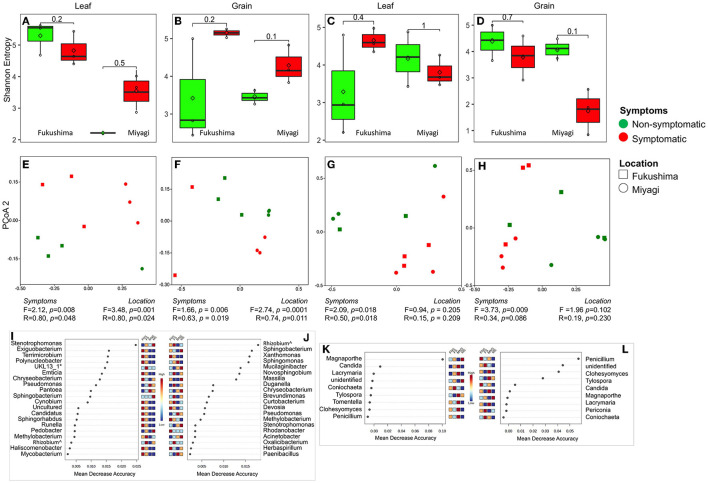
Bacterial and fungal microbiome community of rice leaf and grain as influenced by fungal pathogen infection: Alpha diversity (Shannon Entropy) of bacterial **(A,B)** and fungal **(C,D)** community in leaf **(A,C)** and grain **(B,D)** tissues. PCoA, ANOSIM, and PERMANOVA-based analyses of bacterial **(E,F)** and fungal **(G,H)** microbiome of symptomatic and asymptomatic samples of leaf **(E,G)** and grain **(F,H)** samples of rice collected from two locations in Japan. Random forest analysis results of bacterial (top 20) **(I,J)** and fungal **(K,L)** genera with the highest discriminatory power between symptomatic and asymptomatic tissues of leaf **(I,K)** and grain **(J,L)**. Red fields show a high abundance and blue fields a low abundance of the particular bacterial genera. FNS, Fukushima non-symptomatic; FS, Fukushima symptomatic; MNS, Miyagi non-symptomatic; MS, Miyagi symptomatic.

We were interested in the microbiome community in symptomatic and non-symptomatic tissues (leaf and grain) from the diseased plants. Shannon alpha diversity analysis indicated no substantial differences in the bacterial and fungal communities between symptomatic and non-symptomatic leaf and grain tissues (Figures 2A–D). PCoA analysis and similarity indices (PERMANOVA, ANOSIM) statistics suggested that there remained clear differences in the bacterial and fungal communities of symptomatic leaves and grains from non-symptomatic tissues and that sampling locations significantly influenced community structuring of leaf and grain communities (Figures 2E–H, Supplementary Tables 4, 5). A further inspection of bacterial group differences using random forest analysis revealed that bacteria belonging to certain families were variably present in non-symptomatic or symptomatic tissues. For instance, the symptomatic leaf tissues had higher abundances of *Xanthomonadaceae, Sphingobacteriaceae, Beijerinckiaceae, Microbacteriaceae*, and *Rhizobiaceae*, and the symptomatic grains were dominated by *Sphingomonadaceae, Burkholderiaceae, and Caulobacteriaceae* families. *Enterobacteriaceae* were found to be highly abundant in the non-symptomatic grains in both the location and the non-symptomatic leaf of Miyagi samples and of the Fukushima samples (Supplementary Figure 3). At the genus level, several bacteria were detected in significantly higher abundance in the non-symptomatic leaves in the Fukushima samples, but they were either lowly present or absent in the Miyagi sample. However, consistently higher abundances of *Stenotrophomonas, Pedobacter, Methylobacterium*, and *Rhizobium* were observed in the symptomatic leaves in both locations (Figure 2I). The bacterial genera *Rhizobium, Novosphingobium, Darnella, Brevundimonas, Curtobacterium, Stenotrophomonas*, and *Acinetobacter* had comparatively increased occurrences in the symptomatic grain tissues in both locations. Only *Phenyracillin* was present abundantly in non-symptomatic grain samples irrespective of the sample location (Figure 2J). As for fungi, members of certain families encountered higher in the non-symptomatic tissues. For instance, *Thelephoraceae, Lindgomycetaceae, Atheliaceae, Cantharellaceae*, and *Aspergillaceae* showed increased abundances in leaf and grain non-symptomatic samples (Supplementary Figures 3C,D). The fungal genera, such as *Coniochaeta, Tylospora, Tomentella, Clohesyomyces*, and *Penicillium* were found abundantly present in non-symptomatic tissues in both the leaf and grain samples regardless of the sampling location (Figures 2K,L).

### Functional prediction: Microbiota functional profiling of microbiome of rice

**Figure 3:**
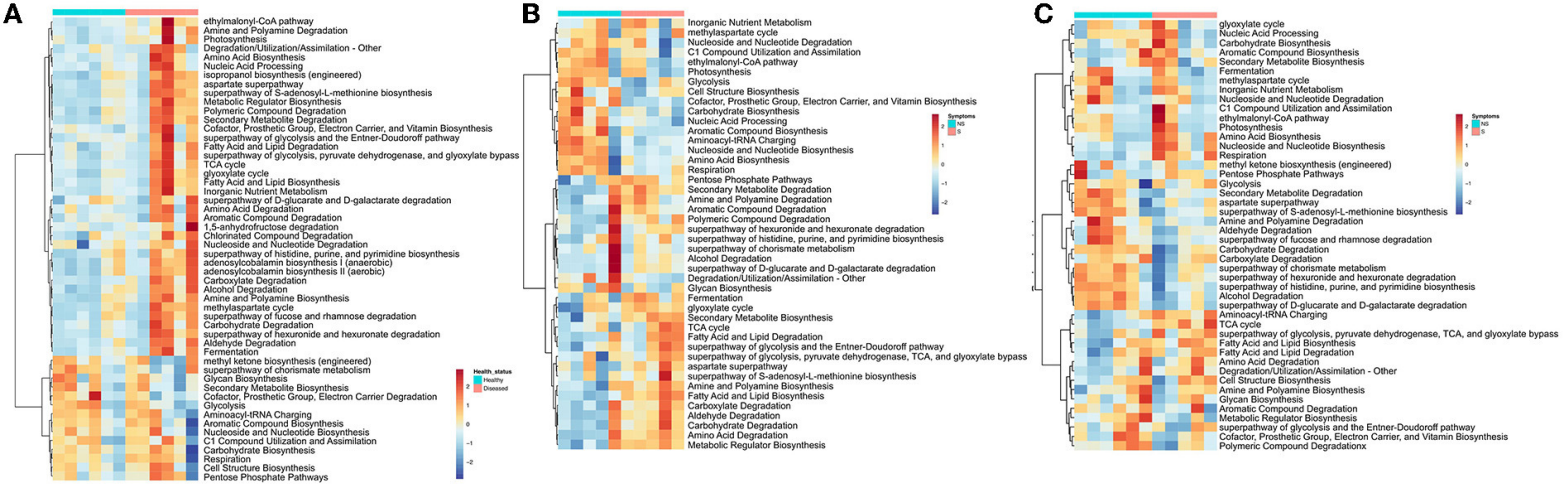
Heatmap Clustering the relative abundance of functional signatures of bacterial microbiome of rice from **(A)** the rhizosphere, **(B)** leaf, and **(C)** grain samples as predicted by Phylogenetic Investigation of Communities by Reconstruction of Unobserved States (PICRUSt2) within Meta-Cyc categories. S and NS represent symptomatic and non-symptomatic tissues, respectively.

The potential metabolic functions of bacterial and fungal communities in symptomatic and non-symptomatic tissues were predicted by PICRUSt2. The partial least squares-discriminant analysis (PLS-DA) plots were determined to evaluate the similarity of the microbial functions between the symptomatic vs. non-symptomatic and diseased vs. healthy samples. The PLS-DA analyses revealed an evident clustering among the samples. There was a difference in the predicted functional pathways of bacterial microbiome communities in diseased rhizosphere and root endosphere (Supplementary Figure 4). The number of abundant functional metabolic pathways predicted from the rhizosphere and root microbiome was higher in diseased plants. For example, “ethylmalonyl-CoA pathway,” “Amine and Polyamine Degradation,” “Amino Acid Biosynthesis,” “isopropanol biosynthesis,” and several other bacterial metabolic pathways were predicted to be abundant in rhizospheres as well as the root endosphere community of diseased plants as compared to healthy plants (Figure 3A; Supplementary Figure 4). Higher abundance of certain other metabolites was predicted in the microbiome rhizosphere of healthy plants, such as “methyleketone biosynthesis,” “Glycan Biosynthesis,” Secondary Metabolite Biosynthesis,” “Cofactor, Prosthetic Group, Electron Carrier, and Vitamin Biosynthesis,” “Glycolysis,” but interestingly, these were comparatively more abundant in the roots of diseased plants (Figure 3A, Supplementary Figure 4). Significant differences in the functional pathways in both bacterial and fungal communities were observed in the symptomatic and non-symptomatic leaf and grain tissues (Figures 3B,C, Supplementary Figure 4). The predicted bacterial functional metabolic pathways, such as “TCA cycle,” “superpathway of glycolysis, pyruvate dehydrogenase, TCA, and glyoxylate bypass,” “fatty acid and lipid biosynthesis,” “fatty acid and lipid degradation,” “amino acid degradation,” and “amine and polyamine biosynthesis” were more abundant in symptomatic tissues in both grain as well as leaf communities (Figures 3B,C). When analyzed the fungal metabolic predictive pathways, certain pathways similar to those of bacterial pathways were found to be more abundant in the symptomatic tissues, such as “fatty acid and lipid degradation” and “amino acid degradation.” The pathways, such as “carbohydrate biosynthesis,” “pentose phosphate pathways,” “TCA cycle,” “inorganic nutrient metabolism,” and “degradation/utilization/ assimilation–other” were more abundant in the fungal metabolites of the non-symptomatic tissues as compared to symptomatic tissues (Supplementary Figure 4). However, some of these pathways predicted from the bacterial community seemed less abundant in the non-symptomatic tissues (Figures 3B,C).

### Network of interactions of *M. oryzae* and other microbial members in the rhizosphere, leaf, and grain microbiome

**Figure 4:**
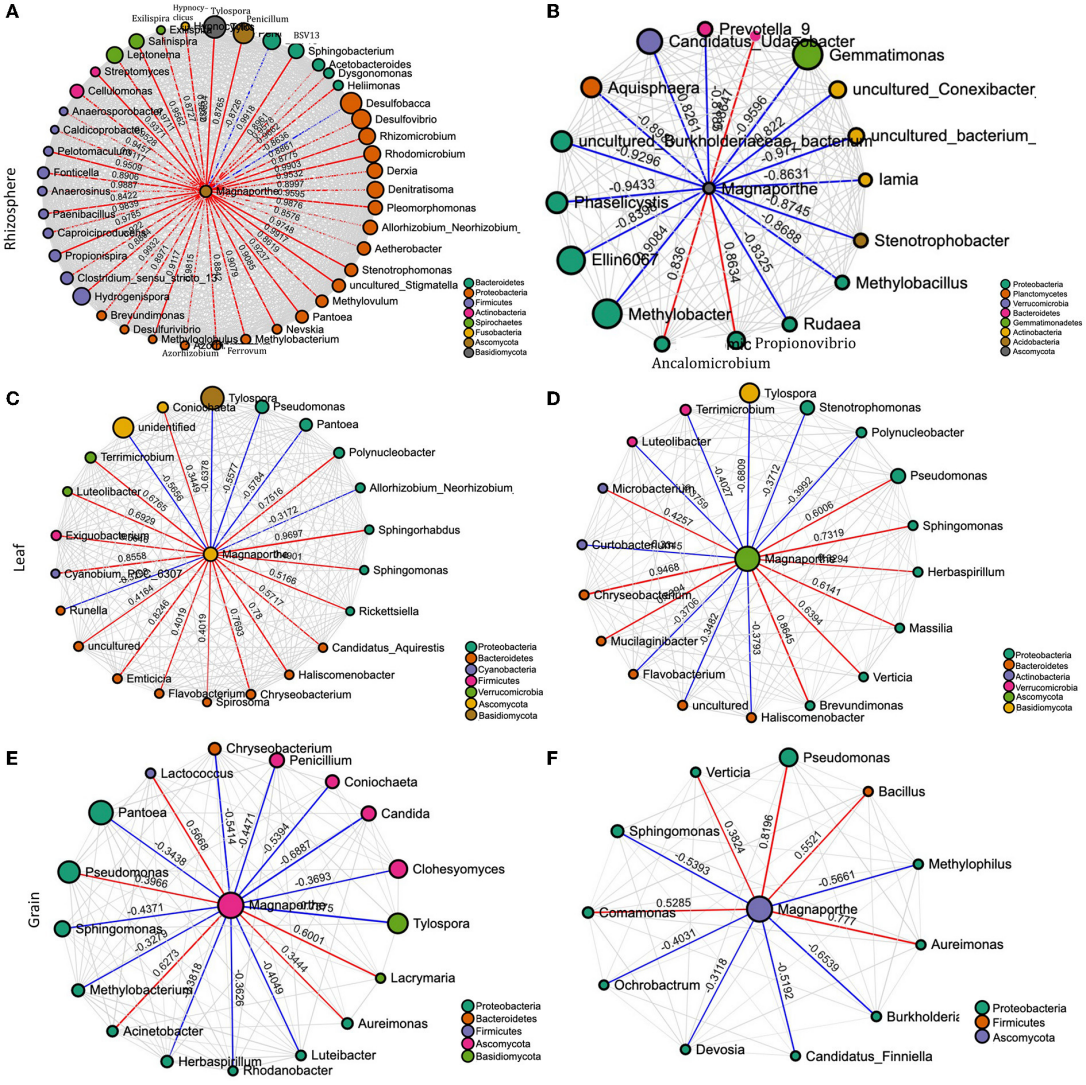
Network of interactions between the pathogen (*M. oryzae*) and other species in the rhizosphere of healthy **(A)** and diseased plants **(B)**, non–symptomatic **(C)** and symptomatic leaf **(D)**, and non-symptomatic **(E)** and symptomatic grain **(F)**. The size of the bubbles corresponds to their relative abundances. The red and blue colors of the edge represent positive and negative interaction with *M. oryzae*, respectively.

To gain a deeper insight into the interactions of *M. oryzae* with other microorganisms, the networks in healthy and infected plants were visualized. The network structures of rhizosphere communities appeared to be significantly altered in *M. oryzae*-infected rice plants showing higher complexity and connectivity than those of healthy plants (Figures 4A,B). We noted more links (43 links) between *Magaporthe* and other microbial genera in the network of infected rhizosphere compartments than the healthy rhizosphere network (17 links), indicating a shift in the rhizosphere microbiome interactions due to foliar infection. The network of interactions in the infected plants revealed that *M. oryzae* were positively correlated with almost all bacterial genera belonging to Bacteroidetes, Proteobacteria, Firmicutes, Actinobacteria, Spirochaetes, and Fusobacteria, and negatively correlated with *Desulfobacca* (Proteobacteria) and a fungal genus, *Penicillium*. In contrast, most bacterial genera were negatively correlated with *M. oryzae* in diseased plant rhizo-microbiome (Figures 4A,B). When we compared the network structures of symptomatic and non-symptomatic tissues, we observed changes in the correlation pattern of *M. oryzae* and other microorganisms (Figures 4C–F). For instance, we noted that there were more links (22 links) between *Magnaporthe* and other microbial genera in the network of non-symptomatic leaf compartments than the symptomatic network (17 links), indicating a shift in the microbiome interactions due to *M. oryzae* infection. Similar was the case in the grain network, where there were more links (21 links) between *Magaporthe* and other microbial genera in the network of non-symptomatic than in the symptomatic network (11 links). Our observations indicate that pathogen invasion alters microbiome members who might assist in colonization through a mutualistic relationship or come to defend the plant against pathogen during the infection process of disease. In other words, microbial members in the non-symptomatic tissues might play roles in keeping the tissues decreased in the invasion of the pathogen that would otherwise cause disease symptoms.

### SourceTracker analysis of microbiome community from soil to other compartments

**Figure 5:**
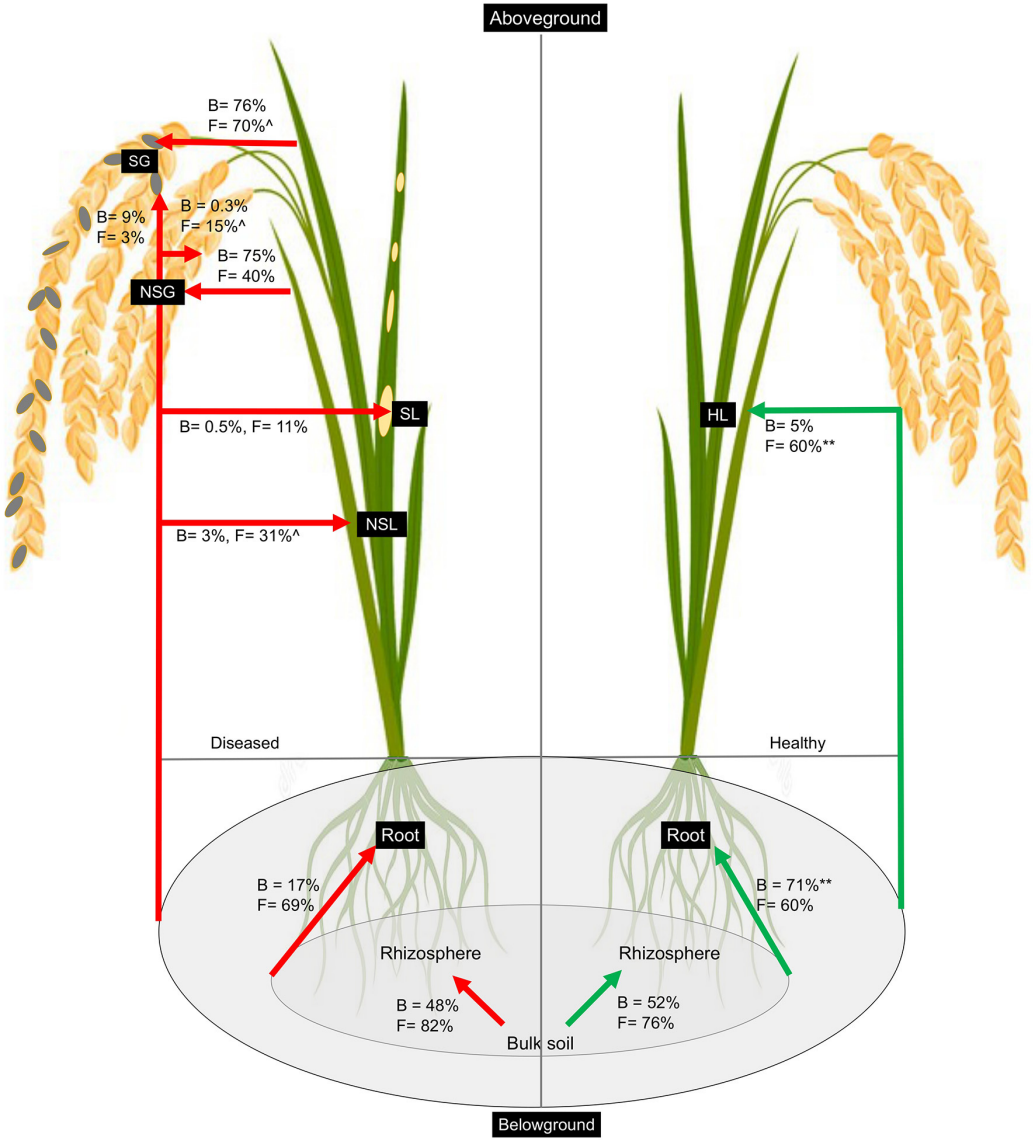
Percentage of bacteria and fungi in each sample that was associated with source types as determined by SourceTracker. The asterisk (**) indicates significant higher percentage at *p* < 0.01 in healthy as compared to diseased samples whereas caret (∧) indicates higher percentage at *p* < 0.05 in non-symptomatic tissues as compared to symptomatic tissues of the diseased plants as obtained from Kruskal–Wallis test.

We utilized the SourceTracker program to study the proportion of bacterial and fungal communities derived from soils. According to the source apportionment results, there were no noticeable differences in the sources of rhizosphere bacterial communities between diseased and healthy plants, and around half of the total were from neighboring bulk soil communities (Figure 5). In healthy samples, the majority of root bacteria community members (71%) were derived from the soil communities (bulk and rhizosphere soils), but rare members (17%) of the root communities of the diseased plant were derived from the soil bacteria communities, indicating there is a clear boundary between the interior and exterior of healthy roots (Figure 5). Very rare members (<10%) of the aboveground (leaf and grain) bacteria were sourced from belowground communities, and the majority were from unknown sources irrespective of the plant health status. Regardless of the symptom expression in grains of diseased plants, most bacteria of gain communities were from leaves (~75%). Regarding the fungal communities, the rhizosphere soil communities were mainly (~80%) derived from the bulk soil in both healthy and diseased plants. More than 60% of root endophytic fungal communities were primarily derived from the soil communities (Bulk and rhizosphere). Notably, more than half (60%) of endophytic leaf communities were derived from the belowground in the soil in the healthy plants, which is significantly less (31%) in the non-symptomatic leaf of the diseased plant and very low (11%) in the symptomatic leaf tissues, indicating most of the aboveground fungal species in the healthy plants could be tracked back from the soils but not in the infected plants. Non-symptomatic grain communities derive 40% of fungi from the leaf and 15% from the belowground. In contrast, symptomatic tissues receive the majority (70%) of fungi from the leaf and are very rare (3%) from belowground communities (Figure 5).

## Discussion

### Rice microbiome composition is shaped by compartments

We present a critical appraisal of bacterial and fungal communities sampled in the bulk soil, rhizosphere soil, the root, leaf, and grain in two different locations of non-symptomatic and symptomatic sample types to provide a comprehensive view of the rice-associated microbiome. Rice-associated bacterial and fungal communities have previously been studied by culture-dependent as well as culture-independent methods (Edwards et al., 2015; Bertani et al., 2016; Kanasugi et al., 2020; Kim and Lee, 2020; Sinong et al., 2020). We profiled the 16S and ITS2 regions to reveal the composition of bacterial and fungal communities. Our results support the concept that distinct plant components play a crucial role in engaging microbial communities, irrespective of the location (Supplementary Figure 1). We demonstrated that the soil (bulk and rhizosphere soil) had a greater diversity of bacterial and fungal communities in the rice plant than endospheric (root and leaf) communities. Bacterial and fungal communities of bulk soil and rhizosphere soil showed no significant difference; however, root endospheric microbial communities differed from leaf communities. The rhizosphere has a diverse and highly populated microbiome and is subject to chemical transformations brought about by the root exudates and metabolites of microbial degradation. The rhizosphere is distinct from the edaphosphere (bulk soil zone), with enhanced microbial activity mainly due to enhanced root exudates deposition (Chaudhary et al., 2012; Reinhold-Hurek et al., 2015; Vieira et al., 2020). However, it is interesting that in our analyses, we observed a slightly higher diversity of rhizosphere bacterial community over bulk soil community in Miyagi but not in Fukushima samples. Although several previous studies reported distinct and more diverse rhizosphere bacterial communities in different crops (Praeg et al., 2019; Hinsu et al., 2021) than in the surrounding bulk soil, we found no distinct separation of the bacterial community of these two zones. This could be due to the fact that the rhizosphere zone is dynamic, and the composition of the microbial community changes as a result of changing root exudation patterns that vary during the life cycle of plants (Edwards et al., 2015; Qu et al., 2020). We collected the samples at the grain maturity or near ripening stage, and microbial diversity is less than the active growth stage of the plant (Hinsu et al., 2021). Previous studies reported that there was no considerable difference in the bulk soil and rhizosphere soil microbial communities. Several others also reported reduced microbiome diversity in rhizosphere communities compared with the bulk soil community (Schlaeppi et al., 2014; Essel et al., 2019). Therefore, in addition to root exudation, the composition of the microbial community in the rhizosphere is linked with the plant developmental stage and several biological and environmental factors (Schreiter et al., 2014; Essel et al., 2019). Our analysis showed that fungal communities were not influenced by the rhizosphere environment. We assume that root exudate deposition might be selective, or bacteria might respond more strongly to some compound secreted by the rice plant. The lower richness and diversity of endophytic communities (root and leaf) compared to the soil (bulk and rhizosphere) samples are understandable. This finding is coherent with the reports from several plant species, including Arabidopsis, soybean, rice, Agave, and others (Knief et al., 2012; Dong et al., 2019). Intuitively, the root and leaf tissues had different microbial communities, and the diversity of bacteria in the leaf was lower than that in the root communities. Similar results were also reported in rice in another study by Wang et al. (2016) and other plant hosts, including Arabidopsis (Bodenhausen et al., 2013), tomato (Dong et al., 2019), and Agave species (Coleman-Derr et al., 2016). The higher bacterial richness in the root compartment may be attributed to the fact that it is the primary site of the interaction of plants with soil and represents one of the richest microbial ecosystems on earth (Hardoim et al., 2011; Tian and Zhang, 2017). It has been reported that the diversity of bacterial endophytes decreased as the distance from soil increased (Ottesen et al., 2013; Dong et al., 2019). However, the reverse pattern was found for the fungal diversity, i.e., higher diversity in the leaf than in the root tissues. The previous study also reported higher diversity of fungi in the shoots than in the roots and attributed to the assumption that most endophytic fungi are derived from aerial fungal spores (horizontal transmission), whereas most of the endophytic bacteria are derived from soils (Rodriguez et al., 2009; Wang et al., 2016).

### Foliar fungal infection shifted bacterial but not fungal microbiome in the rhizosphere

It was found that the blast-infected samples had different bacterial community structures compared to the healthy plants in the root and rhizosphere, but there was no apparent difference in the fungal community (Figure 1). Our study indicated that bacteria of several families were enriched differentially in the rhizosphere and the roots of the healthy and diseased plants. In particular, members of Streptomycetaceae were found to be significantly more abundant in the root endosphere of healthy plants in both locations (Figure 3). The members of Streptomyces are famous for their ability to produce various bioactive compounds and play essential roles in the agricultural field through their biological control potential against phytopathogens, including *M. oryzae* fungi (Procópio et al., 2012; Ser et al., 2016; Law et al., 2017). Endophytic or N-fixing *Burkholderia* strains show immense potential as a biocontrol, as well as plant growth promotion and bioremediation agent (Compant et al., 2008; Elshafie and Camele, 2021). After risk assessment, these *Burkholderia*- products were withdrawn from the market, as some strains may pose a risk to human health. However, over the last several years, the number of new *Burkholderia* species that show plant-beneficial properties and are associated with clinical patients has increased enormously (Eberl and Vandamme, 2016). The *Caulobacter* genus is often isolated from the endosphere or rhizosphere of various plants (Fitzpatrick et al., 2018; Walters et al., 2018; Luo et al., 2019).

The role of plant-associated microbiota in protection against pathogenic fungi and oomycetes is well documented. Although we did not examine empirically, the differential bacterial assemblage may be linked to the differential compound exudation through the rhizosphere due to plant health status responsible for specific microbial group selection, thus regulating their community compositions. The “cry for help” hypothesis suggests that plants recruit microbial partners to maximize their survival and growth when affected by external stress and is likely a survival strategy conserved across the plant kingdom (Liu et al., 2019, 2020; Gao et al., 2021). However, we do not know if the differential microbiome assemblage is the effect of disease or the cause of disease manifestation. Either hypothesis (cause or effect) or even both simultaneously might be possible, and clarification is warranted with manipulative experiments. We detected the presence of *Magnaporthe* in the rhizosphere of healthy samples. Interestingly, the co-occurrence networks analysis of *Magnaporthe* with other taxa revealed that the rhizosphere of healthy plants had a robust and more complex network with a higher number of nodes and edges than the diseased network. Most edges of healthy rhizo-microbiome networks were positively correlated with *Magnaporthe*, whereas they were primarily negative in diseased plants (Figure 4). Again, there were no fungal taxa correlated with *Magnaporthe* in the diseased plant. Only one ascomycetous genera were positively correlated with it, suggesting the putative importance of bacterial taxa in plant health status under *M. oryzae* attack compared to the fungal microbiome. A previous study also highlighted the higher stability of bacterial than the fungal networks in healthy plants. *Fusarium* infection in Chili pepper decreased the complexity of bacterial networks but increased the complexity of fungal networks (Gao et al., 2021). Our functional prediction analysis indicated that the rhizosphere microbiome of diseased plants putatively carries out more diverse metabolic functions (Figure 3) than those associated with healthy individuals, which may be related to disease-induced microbiome recruitment to defend plants under stress. Metaproteomic and transcriptomic analyses, as well as microbiome manipulation of diseased vs. healthy plants, would provide an increased understanding of those microbial community and their functions.

### Pathobiome showed clear separation of the microbiome from those of healthy tissues

The pathobiome concept has been defined as the totality of microbes interacting with a given pathogen species and their influence on pathogenesis (Vayssier-Taussat et al., 2014; Jakuschkin et al., 2016). Characterization of the components of the pathobiome is an important consideration for understanding the pathogenesis, persistence, transmission, and evolution of pathogenic agents (Vayssier-Taussat et al., 2014). Shifts in the composition, richness, and abundance of the microbiome have been shown to occur due to pathogen infection in plants (Jakuschkin et al., 2016; Musonerimana et al., 2020; Mannaa and Seo, 2021). Our results also revealed that the composition of the fungal and bacterial community of rice leaves changed markedly with the *M. oryzae* infection. Through the species classification, we found not only the ASVs assigned to potentially pathogenic *Magnaporthe* were rarely observed abundantly in symptomatic tissues but also from healthy leaf and grain, albeit with significantly low abundances (Figure 2). We could not technically affirm all those ASVs assigned to *Magnaporthe* are pathogenic by just using ITS sequences. The presence of *Magnaporthe* in the healthy tissue implies either there was a latent infection of the pathogen, or these were non-pathogenic strains of *Magnaporthe*. Correspondingly, the relative abundances of some bacteria and fungi were clearly altered in symptomatic tissues. There was a significant increase in the relative abundance of members, such as *Rhizobiaceae, Microbacteriaceae, Beijerinkiacee*, and *Xanthomonadaceae* in the symptomatic over the non-symptomatic tissues. As for fungi, there were higher abundances of *Thelephoraceae, Lingomycetaceae, Atheliaceae, Catharellaceae*, and *Aspergillaceae* families in the non-symptomatic leaves in both locations. These compositional changes could be a cause or consequence of the pathogen invasion. Species of *Paenibacillus* are well-known pathogen suppressors *in planta*. In many studies, several *Paenibacillus* have been identified and used as biocontrol agents with the ability to secrete antibiotics or other antimicrobial proteins and have been applied to prevent and control various plant pathogens, including *Magnaporthe* (Rybakova et al., 2016; Padda et al., 2017; Yu et al., 2019). In the plant endophytic compartments, the relative abundances of *Paenibacillus, Enterobacter*, and several other bacteria that are often considered plant-beneficial microbes showed a significant decrease in abundance compared to the healthy samples. These decreases imply that these endophytic taxa might be excluded due to compromised immune systems locally or outcompeted by more successful colonizers. The shift in the microbiome composition in the symptomatic tissue could be due to the degradation of plant tissue (necrotic/decomposed tissue) by the pathogen that results in colonization with different microbiomes or colonization of bacteria on dead fungal hyphae for their nutrition or bacterial endosymbiont of fungi or pathogen teaming up with commensals (Lundberg et al., 2012; Venturi and da Silva, 2012; Tláskal et al., 2016). On the other hand, the relative abundances of several bacteria, such as those belonging to *Rhizobiaceae, Xanthomonadaceae*, and *Sphingobacteriaceae* in the symptomatic leaf and grain and a few more only in the grain tissues samples showed a marked increase compared to non-symptomatic samples, suggesting they might be involved in the pathogenesis and have mutualistic relationships with the pathogen, or maybe they are opportunists and could take advantage of different ecological niches created by pathogen invasion (Lundberg et al., 2012; Hu et al., 2020). The relatively higher abundances of *Rhizobium* in the symptomatic tissues implied that this strain could either be involved in mutualism with the *M. oryzae* in the disease process or may remain as saprophyte by adopting an oligotrophic lifestyle (Poole et al., 2018). Legume-rhizobium symbiosis is well documented, and much of the rhizobia research has focused on specific parts of the rhizobia–legume symbioses. Our current understanding of rhizobia interaction with non-legumes and its ecology is not comprehensive. Several studies reported rhizobium from the rhizosphere and endosphere of non-legume crops, including rice, and described their roles in plant growth promotion (Peng et al., 2008; Zhang et al., 2014).

According to the inferred microbial network, several direct interactions between *M.oryzae* and other microbes may occur. In contrast to our expectation, the results suggested that mutualism, facilitation, and commensalism may dominate in the interactions of *Magnaporthe* with other microbes in leaves. Almost all the edges in the interaction network were indeed positive in the symptomatic tissues whereas only about half of the edges were positive in the network of healthy tissues. The positive interactions between microbes and the fungal pathogen may be accounted for by endosymbiosis, or they may also be accounted for by commensalism, where bacteria may use dead hyphae as a source of nutrients (Hoffman and Arnold, 2010; Venturi and da Silva, 2012). Bacteria may also facilitate fungal infection because they benefit from the changes in the plant metabolism induced by the fungal pathogen (Venturi and da Silva, 2012). Several *Pantoea* species have been isolated as endophytes without causing symptoms; some isolates are antibiotic producers and have been developed as biocontrol agents for plant diseases (Walterson and Stavrinides, 2015; Jiang et al., 2019). However, this bacterial genus is highly diverse (Walterson and Stavrinides, 2015) and has also been reported to cause disease in rice (Doni et al., 2019). As per the interaction network, there may be a direct, antagonistic (negative) ecological interaction between *Magnaporthe* and *Pantoea* (Figure 4). This finding suggests the putative roles of *Pantoea* to protect against the blast disease. Manipulative experiments are therefore required to validate the isolation and the antagonistic relationship between the two species and decipher the mechanism underlying the interaction.

Soil microbes have the potential to influence plant immune defense and pathogen invasion. Therefore, understanding how to plant endosphere microbes that interact with the soil communities may provide a “road map” to explain the pathogen infection and disease process. Previous studies used the SourceTracker program to estimate the proportions of the microbiome in a given community that comes from potential source environments and have been used to analyze the relationship between disease-associated endosphere microbial communities and rhizosphere communities (Knights et al., 2011; Hu et al., 2020). In this study, we utilized this program to track the source of plant rhizospheric and endospheric microbial communities in healthy vs. diseased plants. Previous studies showed that bacterial communities in the rhizosphere soils were mainly derived from the bulk soil (Mendes et al., 2013; Hu et al., 2020). We found a somewhat difference in that although most fungal communities of the rhizosphere were from bulk, only half of the total bacteria in the rhizosphere were derived from the nearby bulk soils (~80%). There is a sharp contrast between the source of root endosphere bacteria resulting from *Magnaporthe* infection. Whereas the majority (71%) of healthy root bacteria could be tracked from soil (rhizosphere and bulk combined), there was only a very small portion for diseased samples (Figure 5). However, it is not clear about the sources of most microbes, especially bacteria, in diseased plants. We assume that early in the pathogen infection, due to potential host immunity or “cry for help,” as mentioned before, the microbiome was localized prior to our sampling, and the diseased plant could not keep dynamic recruitment of bacteria from soil whereas the healthy individual could. However, controlled experiments with a defined microbiome (synthetic community) and tracking for microbiome sources could reveal important information.

Taken together, we infer that foliar infection by pathogenic *M. oryzae* causes a shift in the rhizosphere bacteria. *M. oryzae* in symptomatic leaf and grain tissues are associated with interactions with various bacteria, including Bacillus, Enterobacter, and several other bacteria, the role of which in the disease process needs to be clarified. The positively interacting bacteria putatively obtain benefits from invading pathogens, which might lead to the migration of many additional bacterial genera into the plant roots and leaves, eventually causing an outbreak of blast disease. These findings will provide potential ideas and a theoretical basis for isolating biocontrol agents controlling the blast disease in rice with further work.

## Data availability statement

The datasets presented in this study can be found in online repositories. The names of the repository/repositories and accession number(s) can be found below: https://www.ncbi.nlm.nih.gov/, PRJNA824966.

## Author contributions

KD: conceptualization, methodology, investigation, formal analysis, software, writing—original draft, and visualization. MY: conceptualization, methodology, writing—review and editing, and project administration. SO: conceptualization, resources, and writing—review and editing, supervision, project administration, and funding acquisition. All authors contributed to the interpretation of the data, critically revised the manuscript, read, and approved the final version before submission.

## Funding

This work was supported by the Japan Society for the Promotion of Science (JSPS) Kakenhi (Grant Number 19H02860).

## Conflict of interest

The authors declare that the research was conducted in the absence of any commercial or financial relationships that could be construed as a potential conflict of interest.

## Publisher's note

All claims expressed in this article are solely those of the authors and do not necessarily represent those of their affiliated organizations, or those of the publisher, the editors and the reviewers. Any product that may be evaluated in this article, or claim that may be made by its manufacturer, is not guaranteed or endorsed by the publisher.

## References

[B1] Anderson M. J. (2014). Permutational multivariate analysis of variance (PERMANOVA), in Wiley Statsref: Statistics Reference Online (Hoboken, NJ: Wiley), 1–15.

[B2] Apprill A. McNally S. Parsons R. Weber L. (2015). Minor revision to V4 region SSU rRNA 806R gene primer greatly increases detection of SAR11 bacterioplankton. Aquatic Microb. Ecol. 75, 129–137. 10.3354/ame01753

[B3] Bäckhed F. Fraser C. M. Ringel Y. Sanders M. E. Sartor R. B. Sherman P. M.. (2012). Defining a healthy human gut microbiome: current concepts, future directions, and clinical applications. Cell Host Microbe 12, 611–622. 10.1016/j.chom.2012.10.012 23159051

[B4] Barrett C. B. (2021). Overcoming global food security challenges through science and solidarity. Am. J. Agric. Econ. 103, 422–447. 10.1111/ajae.12160

[B5] Bass D. Stentiford G. D. Wang H.-C. Koskella B. Tyler C. R. (2019). The pathobiome in animal and plant diseases. Trends Ecol. Evol. 34, 996–1008. 10.1016/j.tree.2019.07.012 31522755 PMC7479508

[B6] Bertani I. Abbruscato P. Piffanelli P. Subramoni S. Venturi V. (2016). Rice bacterial endophytes: isolation of a collection, identification of beneficial strains and microbiome analysis. Environ. Microbiol. Rep. 8, 388–398. 10.1111/1758-2229.12403 27038229

[B7] Beule L. Karlovsky P. (2020). Improved normalization of species count data in ecology by scaling with ranked subsampling (SRS): application to microbial communities. PeerJ 8, e9593. 10.7717/peerj.9593 32832266 PMC7409812

[B8] Bodenhausen N. Horton M. W. Bergelson J. (2013). Bacterial communities associated with the leaves and the roots of *Arabidopsis thaliana*. PLoS ONE 8, e56329. 10.1371/journal.pone.0056329 23457551 PMC3574144

[B9] Bokulich N. A. Kaehler B. D. Rideout J. R. Dillon M. Bolyen E. Knight R.. (2018). Optimizing taxonomic classification of marker-gene amplicon sequences with QIIME 2's q2-feature-classifier plugin. Microbiome 6, 1–17. 10.1186/s40168-018-0470-z 29773078 PMC5956843

[B10] Bolyen E. Rideout J. R. Dillon M. R. Bokulich N. A. Abnet C. C. Al-Ghalith G. A.. (2019). Reproducible, interactive, scalable and extensible microbiome data science using QIIME 2. Nat. Biotechnol. 37, 852–857. 10.1038/s41587-019-0209-9 31341288 PMC7015180

[B11] Bulgarelli D. Schlaeppi K. Spaepen S. Van Themaat E. V. L. Schulze-Lefert P. (2013). Structure and functions of the bacterial microbiota of plants. Annu. Rev. Plant Biol. 64, 807–838. 10.1146/annurev-arplant-050312-120106 23373698

[B12] Busby P. E. Soman C. Wagner M. R. Friesen M. L. Kremer J. Bennett A.. (2017). Research priorities for harnessing plant microbiomes in sustainable agriculture. PLoS Biol. 15, e2001793. 10.1371/journal.pbio.2001793 28350798 PMC5370116

[B13] Callahan B. J. McMurdie P. J. Rosen M. J. Han A. W. Johnson A. J. A. Holmes S. P. (2016). DADA2: high-resolution sample inference from *Illumina amplicon* data. Nat. Methods 13, 581–583. 10.1038/nmeth.3869 27214047 PMC4927377

[B14] Caporaso J. G. Lauber C. L. Walters W. A. Berg-Lyons D. Lozupone C. A. Turnbaugh P. J.. (2011). Global patterns of 16S rRNA diversity at a depth of millions of sequences per sample. Proc. Natl. Acad. Sci. USA 108(Supplement 1), 4516–4522. 10.1073/pnas.1000080107 20534432 PMC3063599

[B15] Caspi R. Billington R. Keseler I. M. Kothari A. Krummenacker M. Midford P. E.. (2020). The MetaCyc database of metabolic pathways and enzymes-a 2019 update. Nucleic Acids Res. 48, D445–D453. 10.1093/nar/gkz862 31586394 PMC6943030

[B16] Chaudhary D. R. Saxena J. Lorenz N. Dick L. K. Dick R. P. (2012). Microbial profiles of rhizosphere and bulk soil microbial communities of biofuel crops switchgrass (*Panicum virgatum* L.) and jatropha (*Jatropha curcas* L.). Appl. Environ. Soil Sci. 2012, 906864. 10.1155/2012/906864

[B17] Chong J. Liu P. Zhou G. Xia J. (2020). Using microbiomeanalyst for comprehensive statistical, functional, and meta-analysis of microbiome data. Nat. Protocols 15, 799–821. 10.1038/s41596-019-0264-1 31942082

[B18] Clarke K. R. (1993). Non-parametric multivariate analyses of changes in community structure. Aust. J. Ecol. 18, 117–143. 10.1111/j.1442-9993.1993.tb00438.x

[B19] Coleman-Derr D. Desgarennes D. Fonseca-Garcia C. Gross S. Clingenpeel S. Woyke T.. (2016). Plant compartment and biogeography affect microbiome composition in cultivated and native Agave species. New Phytol. 209, 798–811. 10.1111/nph.13697 26467257 PMC5057366

[B20] Compant S. Nowak J. Coenye T. Clément C. Ait Barka E. (2008). Diversity and occurrence of *Burkholderia* spp. in the natural environment. FEMS Microbiol. Rev. 32, 607–626. 10.1111/j.1574-6976.2008.00113.x 18422616

[B21] Compant S. Samad A. Faist H. Sessitsch A. (2019). A review on the plant microbiome: ecology, functions, and emerging trends in microbial application. J. Adv. Res. 19, 29–37. 10.1016/j.jare.2019.03.004 31341667 PMC6630030

[B22] Dastogeer K. M. Tumpa F. H. Sultana A. Akter M. A. Chakraborty A. (2020). Plant microbiome-an account of the factors that shape community composition and diversity. Curr. Plant Biol. 23, 100161. 10.1016/j.cpb.2020.10016124871104

[B23] Defazio J. Fleming I. D. Shakhsheer B. Zaborina O. Alverdy J. C. (2014). The opposing forces of the intestinal microbiome and the emerging pathobiome. Surg. Clin. 94, 1151–1161. 10.1016/j.suc.2014.08.002 25440116 PMC4254556

[B24] Devanna B. N. Jain P. Solanke A. U. Das A. Thakur S. Singh P. K.. (2022). Understanding the dynamics of blast resistance in rice-*Magnaporthe oryzae* interactions. J. Fungi 8, 584. 10.3390/jof8060584 35736067 PMC9224618

[B25] Dong C.-J. Wang L.-L. Li Q. Shang Q.-M. (2019). Bacterial communities in the rhizosphere, phyllosphere and endosphere of tomato plants. PLoS ONE 14, e0223847. 10.1371/journal.pone.0223847 31703074 PMC6839845

[B26] Doni F. Suhaimi N. S. M. Mohamed Z. Ishak N. Mispan M. S. (2019). Pantoea: a newly identified causative agent for leaf blight disease in rice. J. Plant Dis. Protect. 126, 491–494. 10.1007/s41348-019-00244-6

[B27] Douglas G. M. Maffei V. J. Zaneveld J. R. Yurgel S. N. Brown J. R. Taylor C. M.. (2020). PICRUSt2 for prediction of metagenome functions. Nat. Biotechnol. 38, 685–688. 10.1038/s41587-020-0548-6 32483366 PMC7365738

[B28] Eberl L. Vandamme P. (2016). Members of the genus Burkholderia: good and bad guys. F1000 Research 5. 10.12688/f1000research.8221.1 27303639 PMC4882756

[B29] Edwards J. Johnson C. Santos-Medellín C. Lurie E. Podishetty N. K. Bhatnagar S.. (2015). Structure, variation, and assembly of the root-associated microbiomes of rice. Proc. Natl. Acad. Sci. USA 112, E911–E920. 10.1073/pnas.1414592112 25605935 PMC4345613

[B30] Elshafie H. S. Camele I. (2021). An overview of metabolic activity, beneficial and pathogenic aspects of *Burkholderia* Spp. Metabolites 11, 321. 10.3390/metabo11050321 34067834 PMC8156019

[B31] Essel E. Xie J. Deng C. Peng Z. Wang J. Shen J.. (2019). Bacterial and fungal diversity in rhizosphere and bulk soil under different long-term tillage and cereal/legume rotation. Soil Tillage Res. 194, 104302. 10.1016/j.still.2019.104302

[B32] Ferreira R. B. Gill N. Willing B. P. Antunes L. C. M. Russell S. L. Croxen M. A.. (2011). The intestinal microbiota plays a role in Salmonella-induced colitis independent of pathogen colonization. PLoS ONE 6, e20338. 10.1371/journal.pone.0020338 21633507 PMC3102097

[B33] Fitzpatrick C. R. Copeland J. Wang P. W. Guttman D. S. Kotanen P. M. Johnson M. T. (2018). Assembly and ecological function of the root microbiome across angiosperm plant species. Proc. Natl. Acad. Sci. USA 115, E1157–E1165. 10.1073/pnas.1717617115 29358405 PMC5819437

[B34] Gao M. Xiong C. Gao C. Tsui C. K. Zhou X. Wang M.-M.. (2021). Disease-induced changes in plant microbiome assembly and functional adaptation. Microbiome 9, 1–18. 10.1186/s40168-021-01138-2 34526096 PMC8444440

[B35] Greenfield M. Pareja R. Ortiz V. Gómez-Jiménez M. I. Vega F. E. Parsa S. (2015). A novel method to scale up fungal endophyte isolations. Biocontrol Sci. Technol. 25, 1208–1212. 10.1080/09583157.2015.1033382

[B36] Hammer Ø. Harper D. A. T. Paul D. R. (2001). Past: Paleontological Statistics Software Package for Education and Data Analysis. Palaeontol. Electron. 4, 9.

[B37] Hardoim P. R. Andreote F. D. Reinhold-Hurek B. Sessitsch A. van Overbeek L. S. van Elsas J. D. (2011). Rice root-associated bacteria: insights into community structures across 10 cultivars. FEMS Microbiol. Ecol. 77, 154–164. 10.1111/j.1574-6941.2011.01092.x 21426364 PMC4339037

[B38] Hinsu A. T. Panchal K. J. Pandit R. J. Koringa P. G. Kothari R. K. (2021). Characterizing rhizosphere microbiota of peanut (*Arachis hypogaea* L.) from pre-sowing to post-harvest of crop under field conditions. Sci. Rep. 11, 1–14. 10.1038/s41598-021-97071-3 34465845 PMC8408145

[B39] Hoffman M. T. Arnold A. E. (2010). Diverse bacteria inhabit living hyphae of phylogenetically diverse fungal endophytes. Appl. Environ. Microbiol. 76, 4063–4075. 10.1128/AEM.02928-09 20435775 PMC2893488

[B40] Hu Q. Tan L. Gu S. Xiao Y. Xiong X. Zeng W.-a.. (2020). Network analysis infers the wilt pathogen invasion associated with non-detrimental bacteria. NPJ Biofilms Microb. 6, 1–8. 10.1038/s41522-020-0117-2 32060424 PMC7021801

[B41] Humphrey P. T. Whiteman N. K. (2020). Insect herbivory reshapes a native leaf microbiome. Nature Ecol. Evol. 4, 221–229. 10.1038/s41559-019-1085-x 31988447 PMC7332206

[B42] Jakuschkin B. Fievet V. Schwaller L. Fort T. Robin C. Vacher C. (2016). Deciphering the pathobiome: intra-and interkingdom interactions involving the pathogen *Erysiphe alphitoides*. Microb. Ecol. 72, 870–880. 10.1007/s00248-016-0777-x 27147439

[B43] Jiang L. Jeong J. C. Lee J.-S. Park J. M. Yang J.-W. Lee M. H.. (2019). Potential of *Pantoea dispersa* as an effective biocontrol agent for black rot in sweet potato. Sci. Rep. 9, 1–13. 10.1038/s41598-019-52804-3 31704990 PMC6841936

[B44] Kanasugi M. Sarkodee-Addo E. Ansong Omari R. Mohammad Golam Dastogeer K. Fujii Y. Oppong Abebrese S.. (2020). Exploring rice root microbiome; the variation, specialization and interaction of bacteria and fungi in six tropic savanna regions in Ghana. Sustainability 12, 5835. 10.3390/su12145835

[B45] Katoh K. Misawa K. Kuma K. Miyata T. (2002). MAFFT: a novel method for rapid multiple sequence alignment based on fast Fourier transform. Nucleic Acids Res. 30, 3059–3066. 10.1093/nar/gkf436 12136088 PMC135756

[B46] Kaushal M. Swennen R. Mahuku G. (2020). Unlocking the microbiome communities of banana (*Musa* spp.) under disease stressed (*Fusarium wilt*) and non-stressed conditions. Microorganisms 8, 443. 10.3390/microorganisms8030443 32245146 PMC7144012

[B47] Kim H. Lee Y.-H. (2020). The rice microbiome: a model platform for crop holobiome. Phytobiomes J. 4, 5–18. 10.1094/PBIOMES-07-19-0035-RVW30812563

[B48] Kirtphaiboon S. Humphries U. Khan A. Yusuf A. (2021). Model of rice blast disease under tropical climate conditions. Chaos Solitons Fract. 143, 110530. 10.1016/j.chaos.2020.110530

[B49] Knief C. Delmotte N. Chaffron S. Stark M. Innerebner G. Wassmann R.. (2012). Metaproteogenomic analysis of microbial communities in the phyllosphere and rhizosphere of rice. ISME J. 6, 1378–1390. 10.1038/ismej.2011.192 22189496 PMC3379629

[B50] Knights D. Kuczynski J. Charlson E. S. Zaneveld J. Mozer M. C. Collman R. G.. (2011). Bayesian community-wide culture-independent microbial source tracking. Nat. Methods 8, 761–763. 10.1038/nmeth.1650 21765408 PMC3791591

[B51] Krezalek M. A. DeFazio J. Zaborina O. Zaborin A. Alverdy J. C. (2016). The shift of an intestinal “microbiome” to a “pathobiome” governs the course and outcome of sepsis following surgical injury. Shock (Augusta, Ga.) 45, 475. 10.1097/SHK.0000000000000534 26863118 PMC4833524

[B52] Krieger C. J. Zhang P. Mueller L. A. Wang A. Paley S. Arnaud M.. (2004). MetaCyc: a multiorganism database of metabolic pathways and enzymes. Nucl. Acids Res. 32, D438–D442. 10.1093/nar/gkh100 14681452 PMC308834

[B53] Law J. W.-F. Ser H.-L. Khan T. M. Chuah L.-H. Pusparajah P. Chan K.-G.. (2017). The potential of Streptomyces as biocontrol agents against the rice blast fungus, *Magnaporthe oryzae* (*Pyricularia oryzae*). Front. Microbiol. 8, 3. 10.3389/fmicb.2017.00003 28144236 PMC5239798

[B54] Liu H. Brettell L. E. Qiu Z. Singh B. K. (2020). Microbiome-mediated stress resistance in plants. Trends Plant Sci. 25, 733–743. 10.1016/j.tplants.2020.03.014 32345569

[B55] Liu H. Li J. Carvalhais L. C. Percy C. D. Prakash Verma J. Schenk P. M.. (2021). Evidence for the plant recruitment of beneficial microbes to suppress soil-borne pathogens. New Phytol. 229, 2873–2885. 10.1111/nph.17057 33131088

[B56] Liu H. Macdonald C. A. Cook J. Anderson I. C. Singh B. K. (2019). An ecological loop: host microbiomes across multitrophic interactions. Trends Ecol. Evol. 34, 1118–1130. 10.1016/j.tree.2019.07.011 31422890

[B57] Louca S. Parfrey L. W. Doebeli M. (2016). Decoupling function and taxonomy in the global ocean microbiome. Science 353, 1272–1277. 10.1126/science.aaf4507 27634532

[B58] Lundberg D. S. Lebeis S. L. Paredes S. H. Yourstone S. Gehring J. Malfatti S.. (2012). Defining the core *Arabidopsis thaliana* root microbiome. Nature 488, 86–90. 10.1038/nature11237 22859206 PMC4074413

[B59] Luo D. Langendries S. Mendez S. G. De Ryck J. Liu D. Beirinckx S.. (2019). Plant growth promotion driven by a novel Caulobacter strain. Mol. Plant-Microbe Interact. 32, 1162–1174. 10.1094/MPMI-12-18-0347-R 30933667

[B60] Ma K.-W. Niu Y. Jia Y. Ordon J. Copeland C. Emonet A.. (2021). Coordination of microbe-host homeostasis by crosstalk with plant innate immunity. Nat. Plants 7, 814–825. 10.1038/s41477-021-00920-2 34031541 PMC8208891

[B61] Mannaa M. Seo Y.-S. (2021). Plants under the attack of allies: moving towards the plant pathobiome paradigm. Plants 10, 125. 10.3390/plants10010125 33435275 PMC7827841

[B62] Martin M. (2011). Cutadapt removes adapter sequences from high-throughput sequencing reads. EMBnet J. 17, 10–12. 10.14806/ej.17.1.200

[B63] Mc Carthy U. Uysal I. Badia-Melis R. Mercier S. O'Donnell C. Ktenioudaki A. (2018). Global food security—issues, challenges and technological solutions. Trends Food Sci. Technol. 77, 11–20. 10.1016/j.tifs.2018.05.00228300759

[B64] Mendes R. Garbeva P. Raaijmakers J. M. (2013). The rhizosphere microbiome: significance of plant beneficial, plant pathogenic, and human pathogenic microorganisms. FEMS Microbiol. Rev. 37, 634–663. 10.1111/1574-6976.12028 23790204

[B65] Musonerimana S. Bez C. Licastro D. Habarugira G. Bigirimana J. Venturi V. (2020). Pathobiomes revealed that *Pseudomonas fuscovaginae* and *Sarocladium oryzae* are independently associated with rice sheath rot. Microb. Ecol. 80, 627–642. 10.1007/s00248-020-01529-2 32474660

[B66] Nguyen N. H. Song Z. Bates S. T. Branco S. Tedersoo L. Menke J.. (2016). FUNGuild: an open annotation tool for parsing fungal community datasets by ecological guild. Fungal Ecol. 20, 241–248. 10.1016/j.funeco.2015.06.006

[B67] Oerke E.-C. (2006). Crop losses to pests. J. Agric. Sci. 144, 31–43. 10.1017/S0021859605005708

[B68] Ottesen A. R. Peña A. G. White J. R. Pettengill J. B. Li C. Allard S.. (2013). Baseline survey of the anatomical microbial ecology of an important food plant: *Solanum lycopersicum* (tomato). BMC Microbiol. 13, 1–12. 10.1186/1471-2180-13-114 23705801 PMC3680157

[B69] Paasch B. C. He S. Y. (2021). Toward understanding microbiota homeostasis in the plant kingdom. PLoS Pathogens 17, e1009472. 10.1371/journal.ppat.1009472 33886694 PMC8061798

[B70] Padda K. P. Puri A. Chanway C. P. (2017). Paenibacillus polymyxa: a prominent biofertilizer and biocontrol agent for sustainable agriculture, in Agriculturally important microbes for sustainable agriculture. (Berlin: Springer), 165–191. 10.1007/978-981-10-5343-6_6

[B71] Patel S. H. Vaidya Y. H. Patel R. J. Pandit R. J. Joshi C. G. Kunjadiya A. P. (2017). Culture independent assessment of human milk microbial community in lactational mastitis. Sci. Rep. 7, 1–11. 10.1038/s41598-017-08451-7 28798374 PMC5552812

[B72] Peng G. Yuan Q. Li H. Zhang W. Tan Z. (2008). *Rhizobium oryzae* sp. nov., isolated from the wild rice Oryza alta. Int. J. System. Evol. Microbiol. 58, 2158–2163. 10.1099/ijs.0.65632-0 18768622

[B73] Poole P. Ramachandran V. Terpolilli J. (2018). Rhizobia: from saprophytes to endosymbionts. Nat. Rev. Microbiol. 16, 291–303. 10.1038/nrmicro.2017.171 29379215

[B74] Praeg N. Pauli H. Illmer P. (2019). Microbial diversity in bulk and rhizosphere soil of Ranunculus glacialis along a high-alpine altitudinal gradient. Front. Microbiol. 10, 1429. 10.3389/fmicb.2019.01429 31338073 PMC6629913

[B75] Price M. N. Dehal P. S. Arkin A. P. (2010). FastTree 2-approximately maximum-likelihood trees for large alignments. PLoS ONE 5, e9490. 10.1371/journal.pone.0009490 20224823 PMC2835736

[B76] Procópio R. E. da Silva I. R. Martins M. K. de Azevedo J. L. de Araújo J. M. (2012). Antibiotics produced by Streptomyces. Braz. J. Infect. Dis. 16, 466–471. 10.1016/j.bjid.2012.08.014 22975171

[B77] Qu Q. Zhang Z. Peijnenburg W. Liu W. Lu T. Hu B.. (2020). Rhizosphere microbiome assembly and its impact on plant growth. J. Agric. Food Chem. 68, 5024–5038. 10.1021/acs.jafc.0c00073 32255613

[B78] Quast C. Pruesse E. Yilmaz P. Gerken J. Schweer T. Yarza P.. (2012). The SILVA ribosomal RNA gene database project: improved data processing and web-based tools. Nucleic Acids Res. 41, D590–D596. 10.1093/nar/gks1219 23193283 PMC3531112

[B79] R Core Team (2020). R: A Language and Environment for Statistical Computing. Vienna, Austria: R Foundation for Statistical Computing.

[B80] Reinhold-Hurek B. Bünger W. Burbano C. S. Sabale M. Hurek T. (2015). Roots shaping their microbiome: global hotspots for microbial activity. Annu. Rev. Phytopathol. 53, 403–424. 10.1146/annurev-phyto-082712-102342 26243728

[B81] Rodriguez R. White J. Jr Arnold A. Redman R. (2009). Fungal endophytes: diversity and functional roles. New Phytol. 182, 314–330. 10.1111/j.1469-8137.2009.02773.x 19236579

[B82] Rybakova D. Cernava T. Köberl M. Liebminger S. Etemadi M. Berg G. (2016). Endophytes-assisted biocontrol: novel insights in ecology and the mode of action of Paenibacillus. Plant Soil 405, 125–140. 10.1007/s11104-015-2526-1

[B83] Santos L. F. Olivares F. L. (2021). Plant microbiome structure and benefits for sustainable agriculture. Curr. Plant Biol. 26, 100198. 10.1016/j.cpb.2021.100198

[B84] Sasse J. Martinoia E. Northen T. (2018). Feed your friends: do plant exudates shape the root microbiome? Trends Plant Sci. 23, 25–41. 10.1016/j.tplants.2017.09.003 29050989

[B85] Schlaeppi K. Dombrowski N. Oter R. G. van Themaat E. V. L. Schulze-Lefert P. (2014). Quantitative divergence of the bacterial root microbiota in *Arabidopsis thaliana* relatives. Proc. Natl. Acad. Sci. USA 111, 585–592. 10.1073/pnas.1321597111 24379374 PMC3896156

[B86] Schreiter S. Ding G.-C. Heuer H. Neumann G. Sandmann M. Grosch R.. (2014). Effect of the soil type on the microbiome in the rhizosphere of field-grown lettuce. Front. Microbiol. 5, 144. 10.3389/fmicb.2014.00144 24782839 PMC3986527

[B87] Sekirov I. Russell S. L. Antunes L. C. M. Finlay B. B. (2010). Gut microbiota in health and disease. Physiol. Rev. 90, 859–904. 10.1152/physrev.00045.2009 20664075

[B88] Ser H.-L. Law J. W.-F. Chaiyakunapruk N. Jacob S. A. Palanisamy U. D. Chan K.-G.. (2016). Fermentation conditions that affect clavulanic acid production in *Streptomyces clavuligerus*: a systematic review. Front. Microbiol. 7, 522. 10.3389/fmicb.2016.00522 27148211 PMC4840625

[B89] Shanahan F. Ghosh T. S. O'Toole P. W. (2021). The healthy microbiome-what is the definition of a healthy gut microbiome? Gastroenterology 160, 483–494. 10.1053/j.gastro.2020.09.057 33253682

[B90] Sinong G. F. Yasuda M. Nara Y. Lee C. G. Dastogeer K. M. Tabuchi H.. (2020). Distinct root microbial communities in nature farming rice harbor bacterial strains with plant growth-promoting traits. Front. Sustain. Food Syst. 4, 314. 10.3389/fsufs.2020.629942

[B91] Skamnioti P. Gurr S. J. (2009). Against the grain: safeguarding rice from rice blast disease. Trends Biotechnol. 27, 141–150. 10.1016/j.tibtech.2008.12.002 19187990

[B92] Smets W. Koskella B. (2020). Microbiome: insect herbivory drives plant phyllosphere dysbiosis. Curr. Biol. 30, R412–R414. 10.1016/j.cub.2020.03.039 32369757

[B93] Song C. Zhu F. Carrión V. J. Cordovez V. (2020). Beyond plant microbiome composition: exploiting microbial functions and plant traits via integrated approaches. Front. Bioeng. Biotechnol. 8, 896. 10.3389/fbioe.2020.00896 32850744 PMC7426627

[B94] Teixeira P. J. Colaianni N. R. Law T. F. Conway J. M. Gilbert S. Li H.. (2021). Specific modulation of the root immune system by a community of commensal bacteria. Proc. Natl. Acad. Sci. USA 118, e2100678118. 10.1073/pnas.2100678118 33879573 PMC8072228

[B95] Tian X.-Y. Zhang C.-S. (2017). Illumina-based analysis of endophytic and rhizosphere bacterial diversity of the coastal halophyte *Messerschmidia sibirica*. Frontiers in microbiology 8, 2288. 10.3389/fmicb.2017.02288 29209296 PMC5701997

[B96] Tláskal V. Voríšková J. Baldrian P. (2016). Bacterial succession on decomposing leaf litter exhibits a specific occurrence pattern of cellulolytic taxa and potential decomposers of fungal mycelia. FEMS Microbiol. Ecol. 92, fiw177. 10.1093/femsec/fiw177 27543318

[B97] Trivedi P. Batista B. D. Bazany K. E. Singh B. K. (2022). Plant-microbiome interactions under a changing world: responses, consequences and perspectives. New Phytol. 234, 1951–1959. 10.1111/nph.18016 35118660

[B98] Turenne C. Y. Sanche S. E. Hoban D. J. Karlowsky J. A. Kabani A. M. (1999). Rapid identification of fungi by using the ITS2 genetic region and an automated fluorescent capillary electrophoresis system. J. Clin. Microbiol. 37, 1846–1851. 10.1128/J.C.M.37.6.1846-1851.1999 10325335 PMC84966

[B99] UNITE Community (2019). UNITE General FASTA Release for Fungi. Version 18.11. 2018. UNITE Community.

[B100] Valent B. (2021). The impact of blast disease: past, present, and future, in Magnaporthe oryzae. (Berlin: Springer), 1-18. 10.1007/978-1-0716-1613-0_1 34236673

[B101] Vannier N. Agler M. Hacquard S. (2019). Microbiota-mediated disease resistance in plants. PLoS Pathog. 15, e1007740. 10.1371/journal.ppat.1007740 31194849 PMC6564022

[B102] Vayssier-Taussat M. Albina E. Citti C. Cosson J. F. Jacques M.-A. Lebrun M.-H.. (2014). Shifting the paradigm from pathogens to pathobiome: new concepts in the light of meta-omics. Front. Cellular Infect. Microbiol. 4, 29. 10.3389/fcimb.2014.00029 24634890 PMC3942874

[B103] Venturi V. da Silva D. P. (2012). Incoming pathogens team up with harmless 'resident' bacteria. Trends Microbiol. 20, 160–164. 10.1016/j.tim.2012.02.003 22390987

[B104] Vieira S. Sikorski J. Dietz S. Herz K. Schrumpf M. Bruelheide H.. (2020). Drivers of the composition of active rhizosphere bacterial communities in temperate grasslands. ISME J. 14, 463–475. 10.1038/s41396-019-0543-4 31659233 PMC6976627

[B105] Walters W. A. Jin Z. Youngblut N. Wallace J. G. Sutter J. Zhang W.. (2018). Large-scale replicated field study of maize rhizosphere identifies heritable microbes. Proc. Natl. Acad. Sci. USA 115, 7368–7373. 10.1073/pnas.1800918115 29941552 PMC6048482

[B106] Walterson A. M. Stavrinides J. (2015). Pantoea: insights into a highly versatile and diverse genus within the Enterobacteriaceae. FEMS Microbiol. Rev. 39, 968–984. 10.1093/femsre/fuv027 26109597

[B107] Wang W. Zhai Y. Cao L. Tan H. Zhang R. (2016). Endophytic bacterial and fungal microbiota in sprouts, roots and stems of rice (*Oryza sativa* L.). Microbiol. Res. 188, 1–8. 10.1016/j.micres.2016.04.009 27296957

[B108] White T. J. Bruns T. Lee S. Taylor J. (1990). Amplification and direct sequencing of fungal ribosomal RNA genes for phylogenetics. PCR Protoc. 18, 315–322. 10.1016/B978-0-12-372180-8.50042-1

[B109] Wickham H. Chang W. Wickham M. H. (2016). Package 'ggplot2'. Create Elegant Data Visualisations Using the Grammar of Graphics. Version 2, 1–189.

[B110] Willing B. P. Vacharaksa A. Croxen M. Thanachayanont T. Finlay B. B. (2011). Altering host resistance to infections through microbial transplantation. PLoS ONE 6, e26988. 10.1371/journal.pone.0026988 22046427 PMC3203939

[B111] Yu W. Q. Zheng G. P. Yan F. C. Liu W. Z. Liu W. X. (2019). Paenibacillus terrae NK3-4: a potential biocontrol agent that produces β-1, 3-glucanase. Biological Control 129, 92–101. 10.1016/j.biocontrol.2018.09.019

[B112] Zhang J. Cook J. Nearing J. T. Zhang J. Raudonis R. Glick B. R.. (2021). Harnessing the plant microbiome to promote the growth of agricultural crops. Microbiol. Res. 245, 126690. 10.1016/j.micres.2020.126690 33460987

[B113] Zhang X.-X. Tang X. Sheirdil R. A. Sun L. Ma X.-T. (2014). *Rhizobium rhizoryzae* sp. nov., isolated from rice roots. Int. J. Syst. Evol. Microbiol. 64(Pt_4), 1373–1377. 10.1099/ijs.0.056325-0 24449787

