# Supplementary material for: Microbiome and pathobiome analyses reveal changes in community structure by foliar pathogen infection in rice
Source: Front Microbiol. 2022 Aug 2;13:949152. doi: 10.3389/fmicb.2022.949152 (PMC9379101; doi:10.3389/fmicb.2022.949152)
Supplement: Supplementary file 1 [file Data_Sheet_1.docx]

Supplementary Material

**Supplementary** **table 1:** Alpha diversity indices values (mean ± SE) (Shannon, Faith’s PD, and Chao1) of bacterial microbiome of healthy and diseased rice samples collected from two locations in Japan. The p-values were obtained by Kruskal-Wallis test.

|  |  |  | Shannon | *p* | Faith’s pd |  | *p* | Chao1 |  | *p* |
| --- | --- | --- | --- | --- | --- | --- | --- | --- | --- | --- |
|  |  | Healthy | Diseased |  | Healthy | Diseased |  | Healthy | Diseased |  |
| Bulk Soil | Fukushima | 10.95+0.15 | 11.182+0.15 | 0.34 | 179.49+11.68 | 203.50+14.49 | 0.267 | 3936.10+449.23 | 4933.47+578.846 | 0.245 |
|  | Miyagi | 10.11+0.03 | 9.96+0.35 | 0.76 | 106.44+2.43 | 103.43+21.84 | 0.43 | 2024.81+149.77 | 1933.60+608.60 | 0.581 |
| *p* |  | **0.021** | **0.032** |  | **0.017** | **0.019** |  | **0.048** | **0.023** |  |
|  |  |  |  |  |  |  |  |  |  |  |
| Rhizosphere | Fukushima | 10.916+0.13 | 10.795+0.07 | 0.483 | 211.62+15.90 | 187.99+10.338 | 0.281 | 4352.17+410.47 | 3844.37+403.07 | 0.427 |
|  | Miyagi | 11.15+0.14 | 10.634+0.39 | 0.321 | 235.14+15.95 | 178.04+34.21 | 0.205 | 5590.27+460.35 | 3564.16+949.39 | 0.127 |
| *p* |  | 0.305 | 0.711 |  | 0.355 | 0.794 |  | 0.115 | 0.799 |  |
|  |  |  |  |  |  |  |  |  |  |  |
| Roots | Fukushima | 6.27+0.12 | 5.55+0.25 | 0.081 | 21.67+0.85 | 17.77+1.11 | 0.050 | 250.08+14.17 | 214.51+30.67 | 0.352 |
|  | Miyagi | 6.96+0.53 | 6.57+0.06 | 0.540 | 31.64+6.03 | 22.77+0.41 | 0.216 | 465.89+113.23 | 273.46+13.41 | 0.167 |
| *p* |  | 0.276 | **0.016** |  | 0.177 | **0.013** |  | 0.132 | 0.153 |  |
|  |  |  |  |  |  |  |  |  |  |  |
| Leaf | Fukushima | 4.18+0.15 | 4.26+0.12 | 0.719 | 4.03+1.31 | 3.81+0.82 | 0.895 | 48.3+5.70 | 75.6+7.75 | **0.047** |
|  | Miyagi | 2.95+0.28 | 2.89+0.24 | 0.880 | 4.14+0.35 | 2.94+0.74 | 0.214 | 11.67+3.18 | 16+6.66 | 0.59 |
| *p* |  | **0.018** | **0.007** |  | 0.940 | 0.473 |  | **0.005** | **0.004** |  |

**Supplementary** **table 2:** Alpha diversity indices values (mean ± SE) (Shannon, Faith’s PD, and Chao1) of fungal microbiome of healthy and diseased rice samples collected from two locations in Japan. The p-values were obtained by Kruskal-Wallis test.

|  |  |  | Shannon |  | Faith’s pd |  | p | Chao1 |  | p |
| --- | --- | --- | --- | --- | --- | --- | --- | --- | --- | --- |
|  |  | Healthy | Diseased |  | Healthy | Diseased |  | Healthy | Diseased |  |
| Bulk Soil | Fukushima | 5.99+0.13 | 5.27+0.62 | 0.24 | 67.05+5.21 | 68.97+19.65 | 0.913 | 316.06+32.23 | 396.54+175.20 | 0.598 |
|  | Miyagi | 4.67+0.15 | 4.35+0.34 | 0.44 | 40.58+4.09 | 53.73+8.22 | 0.225 | 157.57+16.99 | 247.94+51.12 | 0.1687 |
| p |  | **0.003** | 0.245 |  | **0.016** | 0.461 |  | **0.012** | 0.385 |  |
|  |  |  |  |  |  |  |  |  |  |  |
| Rhizosphere | Fukushima | 5.56+0.26 | 5.67+0.13 | 0.73 | 82.52+14.38 | 77.87+10.13 | 0.81 | 403.71+126.22 | 358.62+65.94 | 0.767 |
|  | Miyagi | 4.78+0.24 | 4.07+0.29 | 0.14 | 81.83+9.66 | 59.81+11.45 | 0.216 | 624.37+94.78 | 264.46+84.77 | 0.047 |
|  |  | 0.092 | **0.029** |  | 0.97 | 0.278 |  | 0.235 | 0.395 |  |
|  |  |  |  |  |  |  |  |  |  |  |
| Roots | Fukushima | 3.52+0.18 | 2.85+0.28 | 0.13 | 10.33+1.11 | 6.78+0.50 | 0.043 | 63.00+10.15 | 36.33+7.42 | 0.101 |
|  | Miyagi | 2.39+0.25 | 2.52+0.43 | 0.81 | 9.39+1.96 | 10.81+1.64 | 0.620 | 38.00+1.00 | 53.67+17.03 | 0.527 |
|  |  | **0.035** | 0.477 |  | 0.678 | 0.192 |  | 0.152 | 0.623 |  |
|  |  |  |  |  |  |  |  |  |  |  |
| Leaf | Fukushima | 4.92+0.23 | 4.81+0.46 | 0.86 | 9.93+0.66 | 12.40+1.44 | 0.170 | 105.33 | 116.5+19.5 | 0.670 |
|  | Miyagi | 4.11+0.04 | 4.52+0.52 | 0.56 | 7.30+0.79 | 6.84+0.55 | 0.703 | 68.67+22.18 | 58.00+7.22 | 0.738 |
| p |  | **0.026** | 0.713 |  | 0.064 | 0.070 |  | 0.106 | 0.239 |  |

**Supplementary** **table 3:** One-way PERMANOVA and ANOSIM analyses results of bacterial (blue color) and fungal (red color) microbiome of healthy rice samples collected from two locations in Japan. Bray-Curtis coefficient of community similarity index was computed by permutation of group membership, with 9999 replicates (F=pseudo-F for Permanova, R=Anosim R)

|  | Leaf | Root | Rhizosphere | Bulk Soil |
| --- | --- | --- | --- | --- |
| Leaf | - | F=24.6, *p*=0.002  R=1.00, *p*=0.002 | F=39.0, *p*=0.002  R=1.00, *p*=0.001 | F=14.1, *p*=0.003  R=0.846, *p*=0.002 |
| Root | F=2.2, *p*=0.023  R=0.331, *p*=0.019 | - | F=29.6, *p*=0.002  R=1.00, *p*=0.002 | F=12.1, *p*=0.005  R=0.668, *p*=0.007 |
| Rhizosphere | F=6.05, *p*=0.001  R=0.896, *p*=0.002 | F=5.43, *p*=0.003  R=0.776, *p*=0.002 | - | F=1.9, *p*=0.050  R=0.22, *p*=0.045 |
| Bulk Soil | F=8.20, *p*=0.003  R=0.922, *p*=0.003 | F=7.22, *p*=0.002  R=0.927, *p*=0.003 | F=1.64, *p*=0.117  R=0.133, *p*=0.113 | - |

**Supplementary** **table 4:** Alpha diversity (Shannon, Faith’s PD, and Chao1) of bacterial microbiome of symptomatic and non-symptomatic rice grain and leaf samples of naturally blast infected rice plants collected from two locations in Japan.

|  |  | Fukushima | Miyagi | p (Kruskal Wallis) | Symptoms | Non symptoms | p (Kruskal Wallis) |
| --- | --- | --- | --- | --- | --- | --- | --- |
| Leaf | Shannon | 5.06+ 0.22 | 3.22+ 0.42 | **0.011** | 4.19+0.35 | 4.52+0.80 | 0.29 |
|  | Faith hd | 5.91+0.52 | 2.45+0.77 | **0.033** | 3.79+0.74 | 5.63+1.28 | 0.088 |
|  | Chao1 | 72.03+11.68 | 20.63+5.86 | **0.011** | 47.87+16.63 | 56.85+14.00 | O.394 |
|  |  |  |  |  |  |  |  |
| Grain | Shannon | 4.11+0.61 | 3.88+0.24 | 0.584 | 4.63+0.27 | 3.44+0.36 | **0.028** |
|  | Faith hd | 4.96+1.07 | 3.09+0.53 | **0.273** | 5.12+0.90 | 2.96+0.62 | **0.028** |
|  | Chao1 | 84.06+33.04 | 80.56+21.52 | 0.584 | 100.29+19.47 | 67.04+28.96 | 0.068 |

**Supplementary** **table 5:** Alpha diversity (Shannon, Faith’s PD, and Chao1) of fungal microbiome of symptomatic and non-symptomatic rice grain and leaf samples of naturally blast infected rice plants collected from two locations in Japan.

|  |  | Fukushima | Miyagi | p (location) | Symptoms | Non symptoms | p (symptoms) |
| --- | --- | --- | --- | --- | --- | --- | --- |
| Leaf | Shannon | 3.97+0.47 | 3.99+0.23 | 0.972 | 4.23+0.23 | 3.73+0.43 | 0.310 |
|  | Faith’s PD | 8.97+1.62 | 7.93+1.22 | 0.651 | 8.60+1.06 | 8.18+1.81 | 0.852 |
|  | Chao1 | 87.35+22.99 | 66.18+18.10 | 0.531 | 72.56+16.98 | 79.7+25.34 | 0.830 |
|  |  |  |  |  |  |  |  |
| Grain | Shannon | 4.08+0.31 | 2.90+0.58 | 0.023 | 2.76+0.55 | 4.22+0.22 | 0.008 |
|  | Faith’s PD | 9.52+1.18 | 7.44+1.28 | 0.047 | 6.05+.90 | 10.91+0.55 | 0.001 |
|  | Chao1 | 75.42+14.66 | 57.64+14.07 | 0.029 | 35.83+5.04 | 97.23+6.49 | <0.0001 |

Supplementary Figure 1: Bacterial and fungal microbiome community of healthy rice as influenced by microbiome compartments. Comparison of Shannon divesity index values of bacterial microbiomes among various comparmtents of Fukushima samples (A) and Miyagi samples (B); Principal co-ordinate (PCoA), ANOSIM, and PERMANOVA based analyses of bacterial microbiome of healthy samples of rice collected from two locations in Japan (C). Random forest analysis results of top 20 bacterial class with the highest discriminatory power among microbiome compartments (D). Red fields show a high abundance, blue fields a low abundance of the particular bacterial class. Comparison of Shannon divesity index values of fungal microbiomes among various comparmtents of Fukushima samples (E) and Miyagi samples (F); Principal co-ordinate (PCoA), ANOSIM, and PERMANOVA based analyses of bacterial microbiome of healthy samples of rice collected from two locations in Japan (G). Barplot shows the relative abundance of fungal class in the samples (H). Bray-Curtis coefficient of community similarity index was computed for comparing bacterial community similarity among groups. The ANOSIM statistic *R* values (up to 1) and PERMANOVA *pseudo-F* indicate similarity/dissimilarity between groups obtained by permutation of group membership, with 9999 replicates.

Supplementary Figure 2: Differencial abundance of bacterial microbiome community of rice as obtained by random forest analysis. Random forest analysis results of top twenty bacterial genera with the highest discriminatory power between diseased and healthy samples of rhizosphere and root compartments. Red fields show a high abundance, blue fields a low abundance of the particular bacterial family. FD= Fukushima Diseased, FH= Fukushima Healthy, MD= Miyagi Diseased, MH= Miyagi Healthy.

Supplementary Figure 3 : Differencial abundance of bacterial and fungal microbiome community of rice as obtained by random forest analysis. Random forest analysis results of top bacterial (A, B) and fungal (C, D) family with the highest discriminatory power between symptomatic and non-symptomatic samples of leaf (A,C) and grain (B,D) compartments. Red fields show a high abundance, blue fields a low abundance of the particular bacterial family.

Supplementary Fig 4. Partial least squares-discriminant analysis (PLS-DA) plots of the functional prediction features obtained from picrust2 showing similarity-dissimilarity of the microbial functions between the symptomatic vs. non-symptomatic and diseased vs. healthy rice samples collected from two locations in Japan.
